# Fungal Diversity Is Not Determined by Mineral and Chemical Differences in Serpentine Substrates

**DOI:** 10.1371/journal.pone.0044233

**Published:** 2012-09-20

**Authors:** Stefania Daghino, Claude Murat, Elisa Sizzano, Mariangela Girlanda, Silvia Perotto

**Affiliations:** 1 Department of Life Sciences and Systems Biology, University of Torino, Torino, Italy; 2 Interdepartmental Centre “G. Scansetti” for Studies on Asbestos and Other Toxic Particulates, University of Torino, Torino, Italy; 3 Unité Mixte de Recherche de l'Institut National de la Recherche Agronomique - Lorraine Université ‘Interactions Arbres/Microorganismes’, Centre de Nancy - Champenoux, France; Dowling College, United States of America

## Abstract

The physico-chemical properties of serpentine soils lead to strong selection of plant species. Whereas many studies have described the serpentine flora, little information is available on the fungal communities dwelling in these sites. Asbestos minerals, often associated with serpentine rocks, can be weathered by serpentine-isolated fungi, suggesting an adaptation to this substrate. In this study, we have investigated whether serpentine substrates characterized by the presence of rocks with distinct mineral composition could select for different fungal communities. Both fungal isolation and 454 pyrosequencing of amplicons obtained from serpentine samples following direct DNA extraction revealed some fungal taxa shared by the four ophiolitic substrates, but also highlighted several substrate-specific taxa. Bootstrap analysis of 454 OTU abundances indicated weak clustering of fungal assemblages from the different substrates, which did not match substrate classification based on exchangeable macronutrients and metals. Intra-substrate variability, as assessed by DGGE profiles, was similar across the four serpentine substrates, and comparable to inter-substrate variability. These findings indicate the absence of a correlation between the substrate (mineral composition and available cations) and the diversity of the fungal community. Comparison of culture-based and culture-independent methods supports the higher taxonomic precision of the former, as complementation of the better performance of the latter.

## Introduction

Serpentine sites are ecologically important environments, as they represent biodiversity hotspots with exceedingly high proportion of endemic species associated with their extreme properties. These particular environments are distributed all over the world and are characterized by nutrient deficiency (especially in nitrogen and phosphorous) and magnesium abundance, together with high phytotoxic heavy metal concentrations (e.g. Cd, Ni, Cr, Pb, Co, Zn), low pH and slow rock weathering [1], [2]. All together, these chemical and physical properties determine the so called “serpentine syndrome” [3]. The ecology of plants inhabiting serpentine soils has been well documented, with many plant species showing physiological and ecological mechanisms of stress-tolerance [2].

The ability of serpentine soils to affect the evolution and distribution of organisms others than plants is less well known. Only few studies on the microbial diversity in serpentine sites are available. Bacterial communities from serpentine soils were more similar to each other than they were to communities from geographically close non-serpentine soils [4]. DeGrood and colleagues [5] compared the microbial community composition of a barren, a revegetated and an undisturbed serpentine soil, suggesting that the bacterial community composition of revegetated and undisturbed serpentine soils were similar.

Soil fungi represent a large reservoir of genetic and functional diversity [6], [7]. To our knowledge, however, most studies on fungal diversity in serpentine soils have only considered symbiotic fungi living in association with plants, like arbuscular mycorrhizal [8], [9] or ectomycorrhizal fungi [10]–[15], while little is known about saprotrophic fungi. Previous isolation of saprotrophic fungi from rocks mixed with soil collected in two abandoned asbestos mines [16] revealed three common dominant species, including *Verticillium leptobactrum*, which turned out to be very active in the biodeterioration of asbestos fibres *in vitro* [16], [17], suggesting an adaptation to this substrate.

Serpentinites are ultrabasic rocks (SiO_2_<45%) and are associated with one or more serpentine minerals, which include also asbestos fibrous minerals, divided in two groups: serpentines and amphiboles. Owing to their high metal content, serpentine sites could be a source of metal-tolerant bacteria and fungi that would be suitable for bioremediation purposes. This aspect, together with the ecological peculiarities of the serpentine sites, prompted us to investigate the serpentine fungal community mainly inhabiting rock fragments and debris, for which there is currently little/no information. The main aims of this study were: 1) to compare taxon detection from these substrates by culture-dependent and culture-independent methods (isolation and identification of the dominant culturable fungi, and direct DNA extraction, amplification and sequencing, respectively); 2) to assess whether serpentine debris characterized by different mineral composition could select for different fungal communities. To better understand limitations and biases of using a single DNA barcoding region in the description of fungal diversity [18]–[20], a meta-taxonomic approach using 454 FLX pyrosequencing of ITS1 and ITS2 regions was used, and accompanied by DGGE profiling.

## Results

### Chemical analyses of the serpentine substrates

The four sites (BALA, JOUV, MOMP, VARA, see Materials and Methods section) in the Western Alps were characterized by the occurrence of different asbestos minerals (Table 1). We evaluated the fraction of extractable macronutrients and metals in the samples as well as the C% and N% (Table 1). The amount of C and N in our samples was low and the C/N ratio was similar in all sites. The amount of exchangeble P was low for BALA, MOMP and VARA substrates. Concerning the macronutrients, BALA, JOUV and MOMP samples showed a high Ca∶Mg ratio if compared with literature data concerning serpentine soils [8], [21], [22]. VARA samples showed the highest (P<0.05) amount of Mg and the lowest Ca∶Mg ratio, possibly because of the presence of magnesite minerals in the site, while the highest amount of Ca (P<0.05) was found in MOMP samples. BALA and VARA were characterized by lower amounts of K and Na than the other two substrates (P<0.05). BALA samples contained a significantly higher (P<0.05) amount of Ni and Cu than the other samples. UPGMA analysis of macronutrient and metal availability segregated the VARA samples from the other samples (56% bootstrap support), while JOUV and MOMP samples were clustered together (53% bootstrap; Figure S1A).

**Table 1:** Description of the sampling sites and samples.

Site ID	BALA	JOUV	MOMP	VARA
**Location**	Balangero (Lanzo Valley, Turin)	Jouvenceaux (Susa Valley, Turin)	Mompantero (Susa Valley, Turin)	Confine (Varaita Valley, Cuneo)
**Fibrous minerals**	Chrysotile/balangeroite [60]	Tremolite [61], [62]	Tremolite/antigorite [62]	Chrysotile/carlosturanite [63]
**Site description**	Serpentinite outcrops and debris bearing fibrous veins in a disused asbestos mine recently object of environmental restoration. Sparse metallophytic vegetation on the serpentinite debris [64].	Metric serpentinite boulders lying in grassland and bearing veins with fibrous tremolite and calcite. The surface of the boulders is characterized by deep erosion and crumbling of the fibrous component.	Serpentinite outcrops bearing frequent fibrous antigorite (non-asbestos) veins and little and unfrequent fibrous tremolite veins. Outcrops occur along the excavated sides of a road crossing a montane hardwood forest and xerophytic grasslands.	Serpentinite outcrops and debris bearing chrysotile-carlosturanite-diopside veins in the Auriol disused mine and in the surrounding area, characterized by a sub-alpine coniferous forest [65].
**Sample description**	Rock debris collected from the mining walls and from the ground of mine terraces.	Rock debris scraped off the boulder surface or collected on the ground beneath the boulder.	Rock debris collected from the ground along excavated road sides.	Very fine debris rich of fibres scraped off the mining walls; rock debris collected from the ground.
Ca/Mg	4.4^a^	7.4^b^	6.7^b^	0.04^c^
Ca	435,7±71^a^	733,4±66^b^	932,6±51^d^	117,7±12^c^
K	5,6±1^a^	81,9±8^b^	24,3±3^c^	19,7±6^c^
Mg	98,8±12^a^	99,6±12^a^	139±3^a^	2621,3±414^b^
Na	7,2±0^a^	81,9±7^b^	57,9±17^b^	7,9±0^a^
P	2,14±0^a^	27,49±2^b^	5,06±1^ac^	7,27±0^c^
Cd	0,07±0^a^	0,03±0^ab^	0,09±0^ac^	0,13±0^ac^
Co	3,35±1^a^	0,27±0^b^	5,87±1^d^	3,13±0^a^
Cr	0,47±0^a^	0,36±0^a^	5,21±0^c^	5,23±1^c^
Cu	4,85±0^a^	3,44±0^b^	2,88±0^b^	1,07±0^c^
Fe	73,07±16^a^	22,80±2^b^	62.83±7^a^	88,22±12^a^
Mn	80±27^a^	43,84±5^ab^	144,32±4^c^	17,62±2^b^
Ni	38,82±5^a^	1,13±0^b^	16,12±2^d^	28±3^c^
Zn	3,88±1^a^	3,07±1^a^	2,85±0^a^	2,35±0^a^
C%	0.61±0^a^	1.14±0^b^	1.00±0^b^	1.32±0^b^
N%	0.05±0^a^	0.10±0^b^	0.08±0^c^	0.08±0^c^
C/N	13,15±3^ab^	11,05±1^ab^	12,46±1^abc^	16,44±0^ac^
**pH**	7,56	7	7,4	9,2

Location, presence of fibrous minerals (including asbestos), brief description of the sites and of the samples, extractable fraction of macro- and micronutrients (µg of ions/g of soil ± standard deviation) C%, N%, C/N (the statistical analysis was performed by ANOVA with Tukey as post-hoc test (P<0.05) in order to compare the results obtained for each element from the four substrates), and average pH of the samples.

### Identification of the dominant culturable fungi

Samples of rock debris, in some cases mixed with soil, were collected in order to enrich for fungi associated with the mineral component of the serpentine substrates. Using the dilution plate method, a mean of 15.1, 12.2, 25.5 and 45.2 CFUs/plate were counted for BALA, MOMP, JOUV and VARA respectively. The four most abundant morphotypes in each substrate were identified with dichotomous keys and by sequencing of the ITS region (Table 2). Other morphotypes were usually represented by very few colonies (often less than 1% of total abundance). Such morphotypes were assigned to twelve species. All fungi corresponded to saprotrophic Ascomycetes, with one exception. *Aspergillus fumigatus* was found with a similar abundance (CFU number/total CFU number) and frequency (number of isolation plates containing the species/total number of plates) in three out of four substrates (Table 2). *Cladosporium cladosporioides* and *Penicillium* spp. were isolated from both JOUV and MOMP samples (Table 2). The other species (*Alternaria alternata*, *Epicoccum nigrum*, *Exophiala salmonis*, *Phialophora fastigiata*, *Acremonium* sp., *Phaeosclera* sp., and a sterile mycelium –assigned to Agaricomycetes based on ITS sequencing, see Tables 2 and 3) were instead isolated only from one substrate.

**Table 2:** Fungi isolated by dilution plates.

	species^a^	abundance (%)^b^	frequency (%)^b^
**BALA**	*Agaricomycetes* sp.	14	19
(tot CFUs: 318)	*Epicoccum nigrum*	4	24
	*Aspergillus fumigatus*	6	14
	*Phaeosclera* sp.	4	29
**MOMP**	*Penicillium canescens*	10	33
(tot CFUs: 110)	*Penicillium restrictum*	6	44
	*Aspergillus fumigatus*	5	11
	*Penicillium canescens*	6	22
**JOUV**	*Alternaria alternata*	24	70
(tot CFUs: 255)	*Cladosporium cladosporioides*	26	40
	*Aspergillus fumigatus*	4	20
	*Penicillium* sp.	6	40
**VARA**	*Cladosporium cladosporioides*	1	17
(tot CFUs: 1085)	*Acremonium* sp.	3	12.5
	*Phialophora fastigiata*	27	46
	*Exophiala salmonis*	1	21

a The four dominant morphotypes for each substrate were identified based on their macro- and micro-morphological features and by sequencing of the ITS region of rDNA in the case of sterile mycelia.

b For each taxon the values of abundance (abundance %: CFU number/total CFU number) and frequency (frequency %: number of isolation plates containing the species/total number of plates) are reported.

**Table 3:** Morphological and molecular identification of fungal isolates, compared with the 454 reads databases.

Isolated species		454-ITS1			454-ITS2			
Morphological ID	Molecular ID (GenBank BlastN)	OTU ID (GenBank identification at lowest rank)	sequence matching (bp)	n° reads/OTU	OTU ID (GenBank identification at lowest rank)	sequence matching (bp)	n° reads/OTU	Isolation and detection by 454
*A. alternata*	*Alternaria sp.*	*Alternaria sp.*	164/164	36	*Alternaria alternata*	114/114	10	+
*A. fumigatus*	*Neosartorya sp.*	*Fusarium sp.*	63/68	132	*Aspergillus sp.*	138/139	9	+
*Acremonium* sp.	*Ascomycete sp.*	*Hypocreomycetidae*	136/141	2	*Hypocreales*	125/125	3	+
*C. cladosporioides*	*Cladosporium sp.*	*Cladosporium sp.*	153/155	44	*Capnodiales*	130/131	39	+
*Epicoccum nigrum*	*Dothideomycetes*	*Pleosporales*	138/142	61	*Pleosporales*	110/110	97	+
*Exophiala salmonis*	*Scolecobasidium sp.*	*Scolecobasidium sp.*	220/220	1	*Scolecobasidium sp.*	116/116	6	+
*P. canescens*	*Penicillium sp.*	*Penicillium sp.*	58/58	13	*Penicillium sp.*	133/134	7	+
*P. restrictum*	*Penicillium sp.*	*Penicillium sp.*	164/173	1	*Penicillium brevicompactum*	112/116	1	−
*Penicillium* sp.	*Penicillium sp.*	*Penicillium sp.*	58/58	16	*Penicillium sp.*	121/121	4	+
*Phaeosclera*	*Phoma sp.*	*Phoma sp.*	137/138	4	*Didymellaceae*	109/111	3	−
*Phialophora fastigiata*	*Phialophora sp.*	*Phialophora sp.*	197/199	2	*Phialophora sp.*	109/109	2	+
Sterile mycelium	*Agaricomycetes*	*Coprinellus sp.*	196/209	2	*Agaricomycetes*	111/111	1	+

The morphological identification of the twelve dominant species isolated from the four serpentine substrates was complemented by ITS1-5.8S-ITS2 region sequencing and BlastN analysis (second column). Each sequence was used as a query against the 454 datasets (both ITS1 and ITS2) and the lowest rank identification of the corresponding nearest matching OTU is reported in the third and sixth columns (for ITS1 and ITS2 respectively), together with the bp matching (N of identical bp/bp total lenght of the matching fragment) and the number of sequences supporting the OTU. The last column indicates whether or not (+ or −) the species was detected by both the dilution plate technique and 454 pyrosequencing from the same substrate.

### Analysis of DNA amplicons obtained from the serpentine substrates by 454 pyrosequencing

To collect meta-taxonomic data from the four serpentine substrates, we amplified both the ITS1 and the ITS2 regions of the nuclear ribosomal gene from soil/rock debris extracted DNA. A total of 11,685 reads was obtained for the two ITS regions. After trimming and quality check 4,952 reads for ITS1 and 2,707 for ITS2 were conserved, distributed within the four substrates as shown in the Tables S1 and S2 respectively.

**Figure 1:**
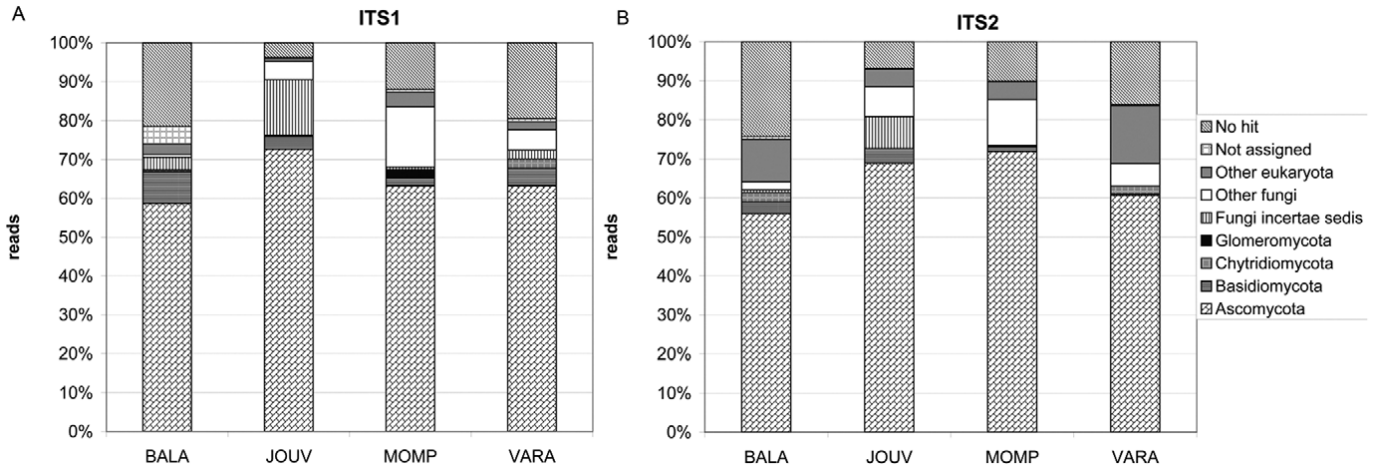
Distribution of the reads within the fungal phyla. The diagram shows the distribution of the sequences obtained by 454 pyrosequencing within the fungal phyla for each substrate, according to the BlastN results in NCBI database (data obtained by MEGAN, [55]).

For ITS1, 82.2% of the total sequences could be assigned to fungi, whereas 75.9% of ITS2 reads could be assigned to this kingdom. This difference is likely due to the fact that the ITS1 region was amplified with primers designed to be fungal specific [23], while the ITS2 region was amplified with universal eukaryotic primers. It is interesting that the universal eukaryotic primers amplified a majority of fungal species in all substrates. The proportion of representatives of the different fungal phyla was approximately the same in all substrates and for both the ITS1 and ITS2 regions (Figure 1), with the dominance of Ascomycota, followed by Basidiomycota. BALA showed more Basidiomycota reads than the other substrates, while JOUV and MOMP showed a higher number of reads corresponding to unidentified fungi (i.e. “fungi incertae saedis” and “other fungi”) than the other two substrates.

**Figure 2:**
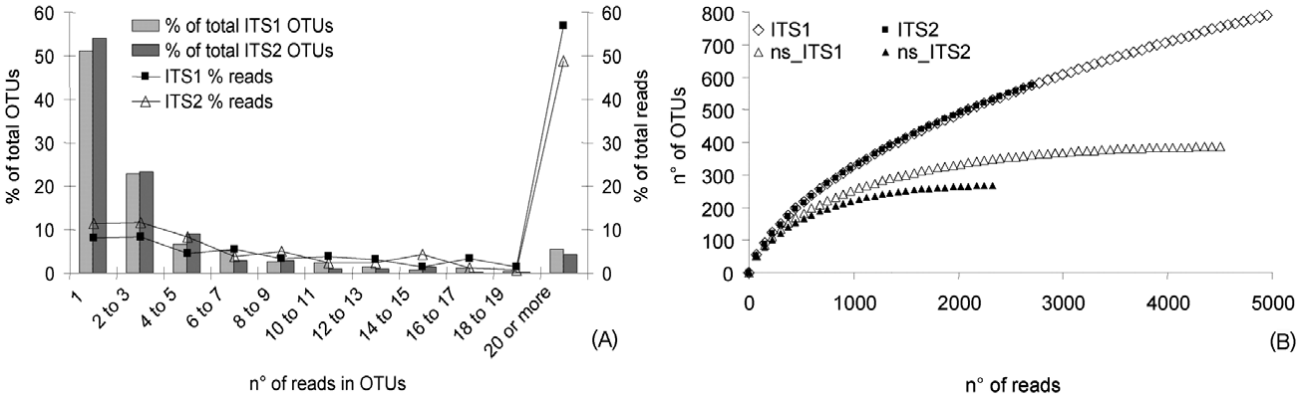
Rarefaction data analysis. (A) Left axis: Clustering of OTUs (as % of total OTUs) according to the number of supporting reads. Right axis: % of the total reads included in each category of OTUs. (B) Rarefaction curves at 97% similarity threshold (OTUs/sampling effort) for ITS1 and ITS2 reads. The rarefaction was calculated both with the total pool of OTUs and with only the non-singletons OTUs.

**Figure 3:**
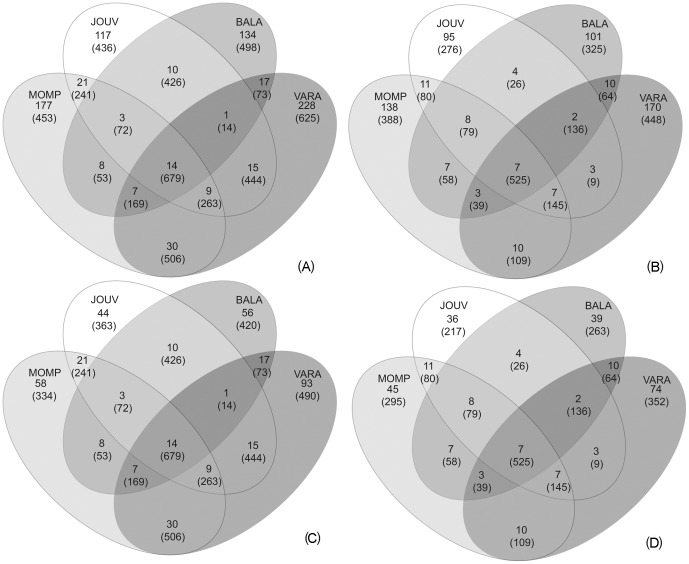
Shared and site-specific OTUs. Venn diagrams representing the distribution of OTUs (and corresponding number of reads in brackets) in the four sampling sites, calculated both considering all the OTUs, and considering only the non-singletons. (A) ITS1 OTUs. (B) ITS2 OTUs (C) ITS1 non-singletons OTUs (D) ITS2 non-singletons OTUs.

The 4,952 ITS1 reads were assembled into 791 OTUs, including 405 singletons, while the 2,707 ITS2 reads were assembled in 576 OTUs, including 310 singletons. The large number of singletons is well illustrated by the OTU clustering curve (Figure 2A), where singletons represented 51% and 54% of total ITS1 and ITS2 OTUs respectively, accounting for 8% and 11% of the total reads. By contrast, 4% and 5% of total ITS1 and ITS2 OTUs, which assembled more than 20 reads each, corresponded to about 57% and 49% of the total reads. When singletons were taken into account, the rarefaction curves for both ITS1 and ITS2 did not reach the plateau (Figure 2B); by contrast, saturation was obtained for both ITS1 and ITS2 when OTUs represented by single reads were not considered. The distribution of OTUs among the four sites (Figure 3) showed that OTUs shared by all substrates comprised a higher proportion of total reads than substrate-specific OTUs. Similarly, OTUs shared by two or three substrates encompassed a considerable fraction of total reads. Indeed, shared OTUs mostly comprised “dominant” OTUs, i.e. OTUs encompassing ≥10 reads. The hierarchical clustering was performed to compare OTU abundances for the four substrates (Figure S1B and S1C). For both ITS1 and ITS2 OTUs, VARA and MOMP samples (which shared the highest number of OTUs, encompassing the highest read number) were clustered together. Analysis of all the ITS1 and ITS2 OTUs segregated the BALA samples from the other samples. However, bootstrap support was low (<60%) in all cases.

Representative sequences of the “dominant” OTUs were used as queries in BlastN searches (Table S3 and S4) and the OTUs that were assigned to the same taxon were grouped together as shown in the tables S1 and S2, reporting the total number of reads supporting each taxon and total abundance for the ITS1 and ITS2 OTUs respectively. For the ITS1 reads, 93 dominant OTUs represented 36 different taxa (Table S1), whereas the 47 ITS2 dominant OTUs corresponded to 27 different taxa (Table S2). Although some OTUs were identified with both the ITS1 and ITS2 rDNA regions, the majority were found only for one region. The most represented orders (*Hypocreales*, *Mortierellales*, *Pleosporales* and *Verrucariales*) were supported by both ITS1 and ITS2 reads, but with different occurrence in the two datasets. The other orders, generally less represented, were often supported by either ITS1 or ITS2 reads (Figure S2).

Most of the dominant OTUs belonged to Ascomycota, but Basidiomycota, Chytridiomycota and Zygomycota were also identified (Tables S1 and S2). Very few taxa could be assigned with confidence to the species level. Within Zygomycota, *Mortierella alpina* was dominant and was recorded by ITS1 in all sites. Within Ascomycota, *Verrucariales*, *Hypocreales*, *Pleosporales*, *Chaetothyriales* and *Capnodiales* were the more represented orders, being detected in all four substrates. Lecanoromycetes fungi represented 5% of ITS1 OTUs and were mainly found in the MOMP and VARA substrates.

### Comparison between culture-independent and culture-based techniques

By comparing culture-dependent and culture-independent methods, we addressed two specific questions: i) how well represented are the dominant fungal isolates in the 454 reads datasets, and ii) how the two methods compare in terms of taxonomic identification. To answer the first question, ITS sequences obtained from the most abundant isolated fungi were used as queries against the ITS1 and ITS2 datasets of pyrosequencing reads. The results based on the nearest matching 454 sequences found are shown in Table 3. Ten out of the twelve taxa isolated in culture were also detected by 454 pyrosequencing in the same site. OTUs matching with isolate sequences were supported by 1 to 132 reads each. Matching of the isolate ITS1 sequence on the 454 pyrosequencing database was in some cases with very short nucleotide strings, but was corroborated by the matching of the ITS2 region, allowing the identification, at least at the genus level (i.e. *A. fumigatus*, *P. canescens* and *Penicillium* sp.). In terms of taxonomic identification, we found in most cases a correspondence between the morphological identification and the BlastN results obtained with both the isolate and the nearest matching 454 sequences, at least at the genus level. In many cases (e.g. *A. fumigatus*, *Acremonium sp.*, *C. cladosporioides*, *Epicoccum nigrum*, *P. canescens*, *P. restrictum*, *Phialophora fastigiata*), morphological identification was more precise than the molecular one, and species-level identification was obtained just based on morphology. For some isolates (e.g. *Exophiala salmonis*, *Phaeosclera sp*), blasting of the ITS sequence(s) yielded consistently a different taxon, if compared with the morphological identification.

**Figure 4:**
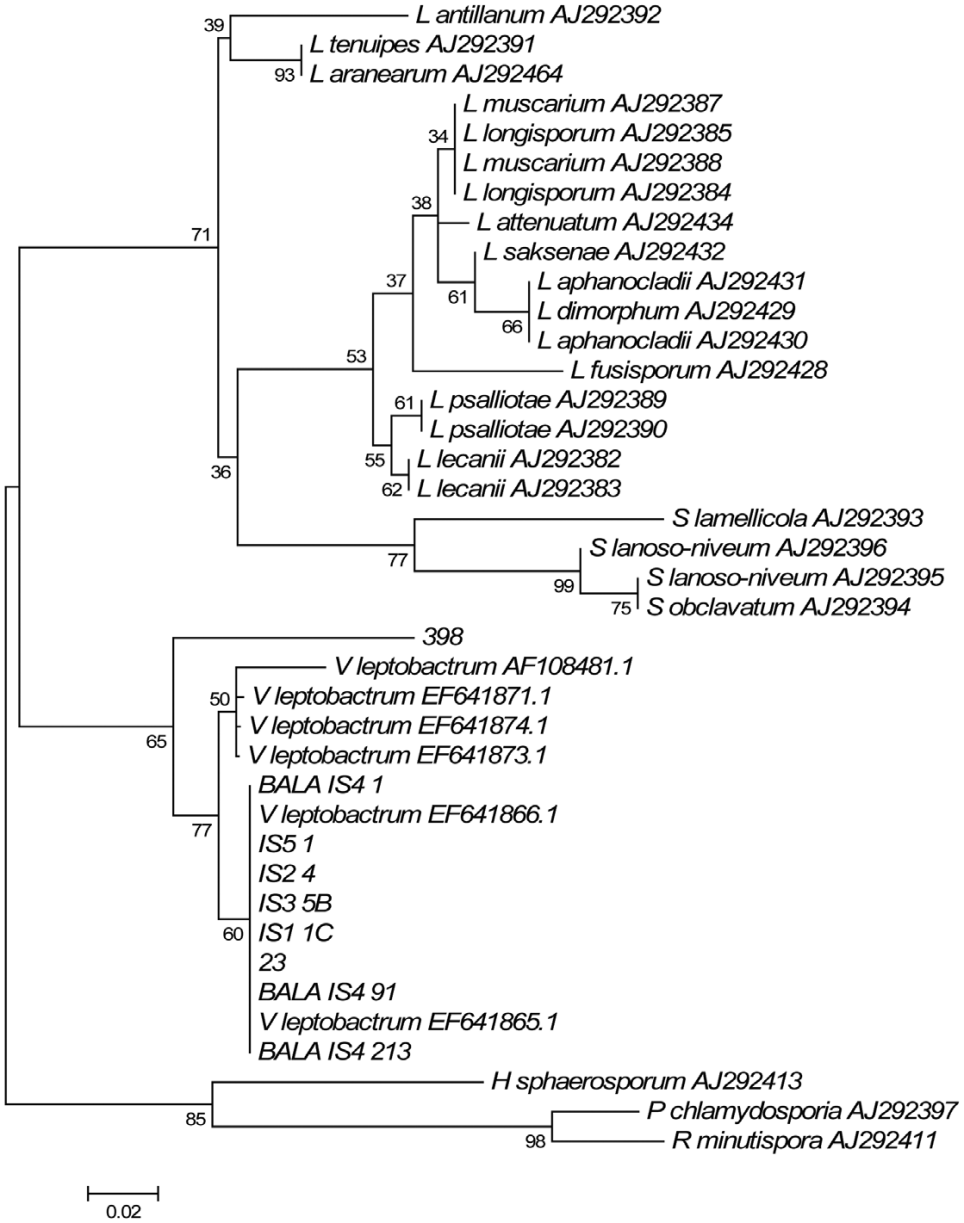
Phylogenetic analysis of *Verticillium* sequences. NJ tree obtained from the ITS2 sequences alignment of 3 *Verticillium leptobactrum* isolates from Balangero site (BALA IS4 91, BALA IS4 213 and BALA IS4 1), 4 sequences of *V. leptobactrum* isolates from other serpentine sites (IS1 1C, IS2 4, IS3 5B and IS5 1; [17]), 7 sequences of *V. leptobactrum*, 17 of *Lecanicillium sp.* and 4 of *Simplicillium sp.* from GenBank and 2 sequences retrieved from the dataset obtained by 454 sequencing (representative of two different OTUs). Sequences of *Rotipherophtora minutispora*, *Haptocillium sphaerosporum* and *Pochonia chlamydosporia* from GenBank were included as out-group. The percentage bootstrap support was calculated out of 1000 trials.

Blast against the 454 pyrosequencing reads was also carried out to check for the occurrence of *Verticillium leptobactrum*, an interesting species that had been previously isolated from the Balangero site [17] but was not found by dilution plates in the current work. Analysis of the BlastN results of all ITS2 OTUs revealed that only one OTU (OTU 23), represented by 21 reads all from the JOUV site, matched with *V. leptobactrum* GenBank sequences within the ten first matches. To investigate the current occurrence of *V. leptobactrum* in the serpentine substrates, and to corroborate the uncertain BlastN results, we used a consensus ITS2 sequence obtained from the previously isolated strains [17] as a query for a Blast search against the dataset containing all the ITS2 sequences from the 454 experiment. We found 9 corresponding OTUs altogether supported by 44 sequences (1 assigned to BALA, 33 to JOUV, 3 to VARA and 7 to MOMP samples). One representative sequence (centroid) per OTU was aligned with reference sequences of *V. leptobactrum* (including 7 sequences of isolates from [17]) as well as with genera closely related to this species (*Lecanicillium*, *Simplicillium*, *Haptocillium*, *Rotiferophtora* and *Pochonia*). Neighbor Joining analysis showed that only two OTUs (OTU 23 and OTU 398), supported by 22 reads (21 and 1 reads respectively) and all from the JOUV site, readily grouped with *V. leptobactrum* sequences. Placement of OTU 23 (which was in agreement with the BlastN results) was supported by 60% bootstrap, whereas OTU 398 (which had been identified only at phylum rank -as *Ascomycota* sp.- by BlastN) clustered as a sister group (with 65% bootstrap support) to the other *V. leptobactrum* sequences (Figure 4). By contrast, sequences of the other OTUs clustered with the *Lecanicillium* and *Simplicillium* sequences (data not shown).

### DGGE analysis of intra- and inter-substrate variability

**Figure 5:**
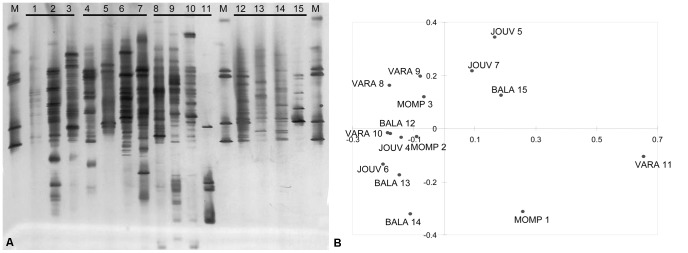
Comparison of the community structure of different serpentine substrates by DNA fingerprinting. (A) DGGE profiles of amplified ITS1 gene fragments from rocks/soil samples (lanes 1-2-3: MOMP, 4-5-6-7: JOUV, 8-9-10-11: VARA, 12-13-14-15: BALA). The gradient of the urea and formamide ranged from 15% to 50%. (B) Principal Coordinate Analysis based on the presence/absence of the amplicons in the DGGE gel. The chart shows that the subsamples of each substrate do not cluster together.

DGGE analysis was carried out to explore the variability in the fungal community within and among substrates. PCoA of the DGGE profiles of the different samples (Figure 5) showed that the different samples from the same substrate were scattered in the ordination space and could be closer to samples from other substrates than to the other samples from the same substrate. Intra-substrate similarity, as expressed by Jaccard similarity coefficients, which ranged 0.048–0.56, did not differ significantly (Kruskall-Wallis test, P = 0.985) among substrates [J_(mean)MOMP_ = 0.31±0.14_(ds)_; J_(mean)JOUV_ = 0.29±0.11_(ds)_; J_(mean)VARA_ = 0.27±0.23_(ds)_; J_(mean)BALA_ = 0.31±0.19_(ds)_]. Overall, Jaccard inter-substrate similarity was low (ranging 0.00–0.60), and did not differ significantly (Mann-Whitney U test, P = 0.984) from overall intra-site similarity [J_(mean)INTRA_ = 0.29±0.16_(ds)_, J_(mean)INTER_ = 0.29±0.15_(ds)_].

## Discussion

### Serpentine substrates harbour diverse fungal assemblages as assessed by culture-dependent and culture independent techniques

In this study, culture-dependent and culture independent techniques were applied to the description of fungal diversity associated to serpentine substrates characterized by the occurrence of different asbestos minerals, macronutrient and metal contents. Isolation and morphological identification of the dominant morphotypes from the four investigated sites mainly yielded *Aspergillus*, *Penicillium* and *Cladosporium* species. These species are in fact ubiquitous saprotrophic fungi easily detected by the dilution plate technique [24]. Among the species isolated from the BALA samples, only *A. fumigatus* had already been retrieved with high abundance in a previous sampling in 2003 [16]. All the species identified have been already isolated from mineral substrates such as limestone, granite, marble, andesite, basalt, gneiss and quartz [25]–[28]. A recent paper investigated fungal diversity in soil samples collected in a Fe-Cu sulphide abandoned mine and containing, among others, serpentine minerals. The fungal community was dominated by *Penicillium* spp., and other isolated species belonged to *Aspergillus*, *Rhizopus*, *Clonostachys*, *Trichoderma* and *Botrytis* genera [29].

As expected, high throughput sequencing of DNA amplicons obtained from the serpentine samples following direct DNA extraction provided a wider fungal spectrum, encompassing both culturable and unculturable taxa. The former included *Mortierella alpina* (Zygomycota), one of the most common saprotrophic soil fungi, not restricted to alpine soil, but with a wide distribution over different climates zones (from Alaska to India) and different kind of soils [24]. Not surprisingly, lichenized fungi such as *Verrucaria* species and Lecanoromycetes were detected among the dominant and common taxa in the rocky substrates that were sampled. *Pleosporales* and *Chaetothyriales*, that are phylogenetically close to several epilithic and endolithic fungi [27], were also detected in all substrates.

Although pyrosequencing of the fungal DNA meta-genome allowed an extensive exploration of the fungal community, molecular identification based on Blast was found to be less precise than morphological identification (Table 3). It is acknowledged that the poor representativeness of public DNA sequence databases, as well as the occurrence of incorrect or incomplete taxonomic annotation, currently prevents accurate identification of the majority of fungal OTUs retrieved by pyrosequencing [30], [31]. In addition, although some 454 OTUs were identified with both the ITS1 and ITS2 rDNA regions, the majority were found only by one region, indicating different taxonomic coverage of the two datasets. Because many of the 454 OTUs identified by a single ITS region were “dominant” OTUs (i.e. OTUs encompassing ≥10 reads), this may be due to the selectivity of the primers rather than to low coverage. These results suggest that the use of both ITS regions in the 454 pyrosequencing experiment provides a better taxonomic coverage than the sole ITS1 or ITS2, in agreement with Bellemain et al. [19] and Mello et al. [32]. Because of the speed and ease of BLAST best-hit analyses, this has become a common approach for ITS sequence-based classifications, although using only the top BLAST hit to classify an unknown sequence is known to be potentially misleading [33]. Porter and Golding [34] have compared the performance of similarity-based (best BlastN hit, MEGAN) or phylogeny-based classification methods of partial and full-length fungal ITS sequences identified by experts (from AFTOL project). Among the tested methods, BlastN was found to have the highest recovery rates (i.e. to yield the largest number of classified queries). However, results from our study and others' indicate that this may not always be the case. In our study, the ITS2 sequences from *Verticillium leptobactrum* isolates matched with nine OTUs otherwise identified, either as close but distinct species or only at genus/order rank, by the BlastN method. In fact, phylogenetic analysis indicated that only two OTUs were actually closely related to this species. Similarly, using simulation to test the performance of methods based either on sequence comparison or on tree topology, Ross and colleagues [35] found that only the tree-based approach was relatively immune to false-positive identifications of species when the query's conspecific sequences were not in the reference dataset. These findings indicate, as already suggested [34], [35], that no single method of species identification is superior across all possible situations, and the strategy for sequence identification should be selected taking into consideration a number of factors, including reference database coverage and an acceptable compromise between the maximum of classifications, and a minimal number of erroneous classifications.

Complementation of 454 pyrosequencing with fungal isolation also allowed us to throw some light on the biological significance of singletons obtained following 454 sequencing. As in other soil meta-taxonomic analyses [6], [36], [37], singletons represented about 50% of the total OTUs. The significance of single read-supported OTUs derived from pyrosequencing is currently under debate. Some authors [7], [38]–[40] consider them as trace of a large unexplored microbial diversity to be considered for richness estimation, whereas Quince and colleagues [41] suggested that Roche's 454 pyrosequencing technology is affected by a noise leading to sequencing errors that contribute substantially, together with chimera formation, to the synthesis of unique reads. Similarly, Tedersoo and colleagues [42], by comparing 454 pyrosequencing and Sanger sequencing results for mycorrhizal fungi, suggested that most of the pyrosequencing singletons were artifactual. The “rare biosphere” would therefore not be as large as estimated by methods (e.g. Chao1 estimator) relying on the number of OTUs that include unique reads [41], [43], [44]. In our work, the ITS regions of some of the fungi isolated in culture matched, in the 454 database, OTUs represented by single reads. This finding indicates that, at least in some cases, single reads correspond to real biological entities.

### Do serpentine substrates characterized by different mineral composition select for different fungal communities?

In this work, both fungal isolation (despite the low number of morphotypes considered) and 454 pyrosequencing revealed some fungal taxa shared by the four serpentinitic substrates, but also highlighted several substrate-specific taxa. The four substrates showed similar C/N ratio, and were all mostly mineral and poorly organic (i.e. they had C% and N% about ten fold lower than those reported for ultramaphic soils [45]), thus indicating that the differences were not determined by a different organic nutrient availability in the substrates. We then investigated a possible correlation with the macronutrient and metal content. Bootstrap analysis (pseudoreplication) of 454 OTU abundances indicated weak clustering of fungal assemblages from the different substrates, which did not match substrate classification based on macronutrient and metal availability. Both PcoA and comparison of Jaccard indices for DGGE profiles showed that intra-substrate variability was similar across the four serpentine substrates, and comparable to inter-substrate variability. These results indicate the absence of a correlation between the substrate (including mineral composition and available cations) and the diversity of the fungal community. Even the VARA samples, that were separated from the other substrates according to the hierarchical clustering (Figure S1A) and were characterized by the lowest Ca∶Mg ratio, one of the challenging features of serpentine soils for plants, did not exhibit particular restrictions or differences in their fungal community.

Overall, our results suggest a low impact of serpentine substrates on the saprotrophic fungal community. Studies on ectomycorrhizal fungi also demonstrated similar communities in nearby serpentine and non-serpentine sites [10], [15], [46]. Greenhouse experiments further showed that the chemical peculiarities of serpentine soils did not limit ECM fungal diversity, and that serpentine communities were neither less diverse than non-serpentine, nor composed of specialized species [11]. Gladish and colleagues [12] found that the serpentine assemblages of ectomycorrhizal fungi from *Pinus* spp. roots were more similar to each other than to non-serpentine ones, but suggested that it is not the soil composition “per se” that determines the mycorrhizal community. Fitzsimons and Miller [8] found a weak effect of serpentine soil properties on the *Avenula sulcata* roots AMF community composition, suggesting that serpentine edaphic factors are less challenging for AMF than they are for plants. On the other hand, Schetcher and Bruns [9] demonstrated that serpentine ecotypes of *Collinsia sparsiflora* associated with a distinct AMF community as compared with non-serpentine ecotypes. Thus, with the exception of the latter, all the papers suggest that the chemical properties of serpentine soils do not limit the diversity of mycorrhizal communities, although it should be noted that mycorrhizal fungi colonise a specialised niche such as plant roots.

We also highlighted temporal changes in the serpentine-associated fungal assemblages. *Verticillium leptobactrum*, a generally rare species, had been isolated from the BALA site in a previous sampling carried out in 2003, where it was unexpectedly very abundant, reaching 27% of isolates [16]. It was therefore surprising, in this study, that no isolates belonging to this species were identified in the BALA samples by dilution plates. Similarly to isolation, 454 sequencing indicated a reduction of *V. leptobactrum* in the Balangero soil to undetectable levels at the moment of the second sampling. Similar results were obtained for the other formerly dominant species [16], with the exception of *A. fumigatus* (detected by dilution plate among the dominant species) and *Mortierella alpina* (detected by pyrosequencing among the dominant species). In the two years from the first sampling, the Balangero site, the largest disused asbestos mine in Europe, has been subjected to a large environmental restoration project involving new plantations to restore the plant coverage, and settling of sides rich of asbestos-bearing rocks debris. Our results suggest that human intervention on this site may have influenced fungal biodiversity by exerting an impact on rare and specialised species, such as *V. leptobactrum*. It is indeed reported that disturbance of native communities provides inroads for invasive species [47].

### Conclusions

Aims of our work were to investigate the dominant/abundant taxa in fungal communities dwelling in serpentine sites, and to reveal possible differences in fungal communities associated with substrates with different mineral and chemical composition, by combining culture-dependent and culture-independent methods. Our results provide a first meta-genomic description of saprotrophic fungal diversity in serpentine substrates. Fungal diversity and distribution were found to be independent of substrate composition. Complementation of 454 pyrosequencing with fungal isolation indicated that OTUs represented by a single read may correspond to real biological entities. Results on *Verticillium leptobactrum* suggest that rapid changes in serpentine-associated fungal communities may occur following disturbance.

## Materials and Methods

### Sites description

Samples were collected in two disused asbestos mines and in two pristine ophiolitic sites with serpentine rocks and fibrous asbestos outcrop occurring in the Western Alps (Ultrabasic Massif of Lanzo and the Piedmont Zone of Calcschist with Metaophiolites, Table 1). The four sites were: the Balangero mine (BALA; Lanzo Valley, Turin, Italy), Mompantero (MOMP; lower Susa Valley, Turin, Italy), Jouvenceaux (JOUV; higher Susa Valley, Turin, Italy), Confine (VARA; Varaita Valley, Cuneo, Italy). Serpentine asbestos was present in two (BALA and VARA) out of four field sites and was represented by chrysotile mineral, a hydrated magnesium silicate where Fe^2+^ is often substitute for Mg^2+^. In these sites, either balangeroite or carlosturanite, two fibrous minerals both hydrated silicates of Fe^2+^, Mn^2+^ and Mg^2+^, were found in association with chrysotile. Antigorite is a serpentine mineral found in the MOMP site, with the same chemical composition of chrysotile, but different mineral structure. Tremolite, outcropping in the JOUV site, is an amphibole containing Ca^2+^, Mg^2+^ and Fe^2+^.

### Sampling and experimental design

The substrates studied were rock fragments or debris containing the different asbestos fibres. These substrates were mostly taken or scraped off rock walls and boulder surfaces. In some cases, rock fragments were also collected on the ground, with some mixed soil. In these cases, although no plants were growing in the sampling points, we cannot exclude the presence of root pieces in the samples (see Table 1 for details).

We collected 15 substrate samples in total: they were taken from four random points in the BALA, JOUV and VARA sites, and from three random points in the MOMP site.

To reduce the bias in DNA extraction, three aliquots were separately extracted for each substrate sample (45 DNA extractions in total, 12 for BALA, JOUV and VARA, 9 for MOMP). Similarly, three aliquots per sample were used for fungal isolation by the dilution plate method (45 isolation plates in total, 12 for BALA, JOUV and VARA, 9 for MOMP).

DNA extracted from the same substrate sample was pooled before PCR amplification, yielding 15 samples for both DGGE analysis and 454 pyrosequencing. For the pyrosequencing experiment, amplicons obtained from the same site were pooled together.

For the chemical analyses, equal amounts of rock fragments or debris derived from all samples collected in each site were mixed, and four separate pools were prepared. For each pool, three separate aliquots were used for pH, C/N, P and cations measures (i.e. three replicates per site).

No specific permits were required for the field studies in the MOMP, VARA and JOUV sites, which are neither privately-owned nor protected. All necessary permits were obtained for sampling in the BALA site, managed by R.S.A that accorded the permission in the frame of the project “Asbestos hazard in Western Alps” lead by the Interdepartmental Centre “G. Scansetti” for Studies on Asbestos and Other Toxic Particulates. The field studies did not involve endangered species in any site.

### Chemical analyses and metal content determination

Equal soil/rock debris aliquots (5 g) from the four (three for MOMP) samples collected in each site were pooled, and chemical analyses were performed on three separate extractions. pH was measured in CaCl_2_ 0.01 M [5], [48]. Phosphorous (Olsen) was extracted and measured by standard procedures [48], and C% and N% were measured with a CE NA 2100 apparatus. Exchangeable cations were extracted with neutral ammonium acetate (NH_4_C_2_H_3_O_2_ 1 N, pH 7; [5], [47] while acid ammonium acetate (NH_4_C_2_H_3_O_2_ 0.5 M, acetic acid 0.5 M, EDTA 0.02 M, pH 4.65; [49]) was used for metal extraction. Both macronutrients and metals were quantified by ICP-AES performed using a Liberty 100 Varian apparatus equipped with a V-Groove nebulizer and a Czerny–Turner monocromator (Department of Mineralogical and Petrological Science, University of Torino) and the results are reported in the Table 1. The statistical analysis was performed by ANOVA with Tukey as post-hoc test. A hierarchical clustering analysis (UPGMA, Euclidean distance as resemblance measure, 100 bootstrap replicates) of the cations extracted from the pool of samples from each site was performed using SYN-TAX 2000 [50].

### Identification of fungi isolated by dilution plates

Fungi were isolated by the dilution plate method. Soil/rock debris aliquots (1 g) from the collected samples were suspended in sterile water (1∶500 g mL^−1^), while fragments of serpentine rocks (approximately the same surface per sample) were washed in sterile water by vortexing for 15 min. A total of 45 aliquots (three per sample) were used to prepare the corresponding suspensions that were plated on 2% MEA [Malt Extract Agar: 20 g L^−1^ malt extract (Merck), 18 g L^−1^ agar (Sigma)], amended with antibiotics [40 mg L^−1^ gentamycine (Essex Italia), 30 mg L^−1^ streptomycin (Sigma)], and were subsequently incubated at room temperature for one-two weeks. Developing fungal colonies were counted (as colony forming units, CFUs), transferred individually into culture tubes and stored at 4°C. The dominant fungi were identified by their morphological characters with dichotomous keys [24]. Sequencing of the Internal Transcribed Spacer (ITS) of the nuclear rDNA region was also carried out, following PCR amplification with the universal fungal primers ITS1F and ITS4 (Table S5) as described in Murat et al. [51]. The sequences obtained were compared to the GenBank [52] database using the BlastN algorithm [53]. The same sequences were also used as queries to find the nearest match in the ITS1 and ITS2 sets of pyrosequencing reads (see below).

### Direct DNA extraction and 454 pyrosequencing

To reduce the bias in DNA extraction, three separate DNA extractions were carried out for each of the four (three for MOMP) samples. Aliquots (0.5 g each) of mixed rock debris and rock fragments were used, the latter after accurate manual grinding with mortar and pestle after immersion in liquid nitrogen. Total DNA extraction was performed by the Fast DNA Spin Kit for Soil (Q-BIOgene, Heidelberg; Germany), using the protocol modified by Luis et al. [54]. DNA obtained from the three replicates was pooled for each sample before PCR amplification, both for 454 sequencing and for DGGE analysis (see below).

The extracted DNA was amplified using two different primer pairs fused with two different 454 pyrosequencing adapters (A and B, Table S5). The first primer pair ITS1F-ITS2 was designed to amplify specifically the fungal ITS1 region (*c.* 400 bp), while the second set ITS3-ITS4 amplifies the ITS2 region (*c.* 350 bp) of eukaryotes [23], [55]. To discriminate among the samples, that were sequenced together, tagged primers were designed (Table S5). The PCR mix contained RedTaq (Sigma) 0.4 U, buffer 10×, primers 0.2 µM, dNTPs 0.2 mM and 2 µl of soil DNA extract. The PCR cycle was: 95°C (4′); 94°C (45″) 50°C (30″) 72°C (1′) X35; 72°C (5′); 10°C hold. Four (three for MOMP) PCR reactions were carried out separately on the different samples from each site and then pooled per site before pyrosequencing.

As the objective of this work was to compare dominant/abundant components of the different fungal communities, rather than to exhaustively describe species diversity, pyrosequencing was carried on two 1/16^th^ 454 GS-FLX runs (instead of the complete plate) by BMR Genomics (Padova, Italy). The reads were submitted to the Sequence Read Archive (SRA) of EBI-EMBL and are available at the following URL http://www.ebi.ac.uk/ena/data/view/ERP001462. The ITS1 and the ITS2 reads obtained from the four sites were extracted with Flower to obtain .fna and .qual files from .sff files. The reads were then filtered for quality (phred 20) and length (>200 bp) with trimseq and all reads with ambiguous bases were discarded. Then ITSextractor was used for isolating ITS1 and ITS2 sequences and to cut the 18S, 5.8S and 28S regions. All the remaining sequences were used for taxonomic identification by comparison against GenBank using the BlastN algorithm. As the library used for the BlastN search can influence the final result [6] we excluded poorly identified sequences by restricting the library to 589,317 sequences corresponding to a Boolean research on NCBI (Eukaryota[organism] AND ribosomal NOT fungal sp. NOT Ectomycorrhiza of NOT uncultured NOT unknown NOT mRNA NOT environmental sample NOT mycorrhiza of NOT endomycorrhiza of NOT *Homo sapiens* NOT unidentified). This 589,317 sequences library was used for BlastN searching of the reads obtained by pryosequencing. MEGAN v.3.7.2 (MEtaGenome ANalyzer, Center for Bioinformatics, Tübingen, Germany; [56]), which provides unique names and IDs for about 700.000 taxa from the NCBI taxonomic database, was used to assign all the reads to the fungal phyla (Figure 1) after BlastN. All parameters of MEGAN, including the lowest common ancestor (LCA) assignment, were kept at default values, except for the “min support” option (regulating the minimum number of sequence reads that must be assigned to a taxon), which was set to one. To assess species richness in the four serpentine sites, Operational Taxonomic Units (OTUs) were generated with the program cdhit (97% sequence identity, [37]) with all reads coming for the four sites. A tag was used in the name of the reads of each site to generate a matrix corresponding to the presence/absence of OTUs in the sites. This matrix was used to generate Venn diagrams. A fasta-formatted sequence file containing only the centroid sequence for each OTU was used for taxonomic identification by the BlastN algorithm (Identity>91%, e-value<E-7). As queries often matched different Blast hits with the same e-value, we considered the first ten blast hits to assign each OTU to the lowest taxonomic rank [57] common to all of them.

A hierarchical clustering analysis (UPGMA, chord distance as resemblance measure, 100 bootstrap replicates) was performed with SYN-TAX 2000 [49] to compare OTU representation in the different sites.

### Phylogenetic analysis focused on Verticillium leptobactrum

In previous work, *Verticillium leptobactrum* was identified, by the dilution plate, as being an abundant species in the Balangero site [17]. This rare and mostly uncharacterized species was found to be extremely active as bioweathering agent against asbestos fibres [16], [17]. To assess detection of this fungus by the 454 approach, sequences generated in the previous study were used to build a phylogenetic tree, together with reference sequences from NCBI and with *V. leptobactrum*-putative sequences retrieved from the 454 database. The sequences were aligned using ClustalW and the alignments were manually corrected. A Neighbour Joining analysis with Kimura-2-parameter was performed with 1000 bootstrap replications with MEGA 3.1 [58].

### DGGE fingerprint analysis

DGGE analysis was carried out to explore the variability in the latter communities within and among substrates. The DGGE technique provides indeed a relatively rapid and inexpensive means to study and compare the community structure of different substrates [59].

The rDNA ITS1 region was amplified from each DNA sample with the ITS1F and ITS2 primers, the former with GC clamp for the subsequent denaturing gradient gel electrophoresis (DGGE) analysis (ITS1F-CG in Table S5). DGGE was performed on the ITS1 amplicons using the DCode™ DGGE system (Bio-Rad). A 1.5 mm thick gel with 15%–50% denaturing gradient and 8% polyacrylammide was prepared for the separation of the approximately 400 bp ITS1 amplicons, where 7 M urea and 40% formamide is defined as 100%. For each sample, 35 µl were loaded on the gel and separated for 17 hours at constant voltage (60 V) and 60°C. The gel was silver stained, and the DGGE profiles of the different samples (Figure 5A) were transformed in a presence-absence (1)-(0) matrix by visual observation. Jaccard similarity coefficients were calculated (SPSS Statistics 17.0) for each pair of lanes on the gel. Intra-site values (three Jaccard indices for Mompantero, six values for the other sites) were statistically compared (Kruskall-Wallis test, performed with SYSTAT 11). The overall intra-site similarity (21 indices) was also compared with the overall inter-site similarity (84 indices) (Mann-Whitney U test, SYSTAT 11). The binary matrix was also used to perform a Principal Coordinate Analysis (PCoA) with XLSTAT for Excel (Figure 5B).

## Supporting Information

Figure S1 **Hierarchical clustering representing the distance among the four substrates.** The clustering was based on (A) the extractable fraction of cations, the distribution and representation of (B) ITS1 OTUs and (C) ITS2 OTUs within the four sites. See Materials and Methods section for the details of the analyses.
(TIF)

Figure S2 **ITS1 and ITS2 blast results at genus level.** The OTUs≥10 reads were grouped according to their taxon assignment at genus level and the number of ITS1 and ITS2 reads supporting each taxon is reported.
(TIF)

Table S1 **Dominant taxa according to the ITS1 region sequencing.**
(DOC)

Table S2 **Dominant taxa according to the ITS2 region sequencing.**
(DOC)

Table S3 **ITS1 dominant OTUs.** OTUs supported by at least 10 reads were ranked according to their abundance.
(DOC)

Table S4 **ITS2 dominant OTUs.** OTUs supported by at least 10 reads were ranked according to their abundance.
(DOC)

Table S5 **Primers used in the described experiments.**
(DOC)
