# Supplementary figures and images for: Fungal Diversity Is Not Determined by Mineral and Chemical Differences in Serpentine Substrates
Source: PLoS One. 2012 Sep 20;7(9):e44233. doi: 10.1371/journal.pone.0044233 (PMC3447857; doi:10.1371/journal.pone.0044233)

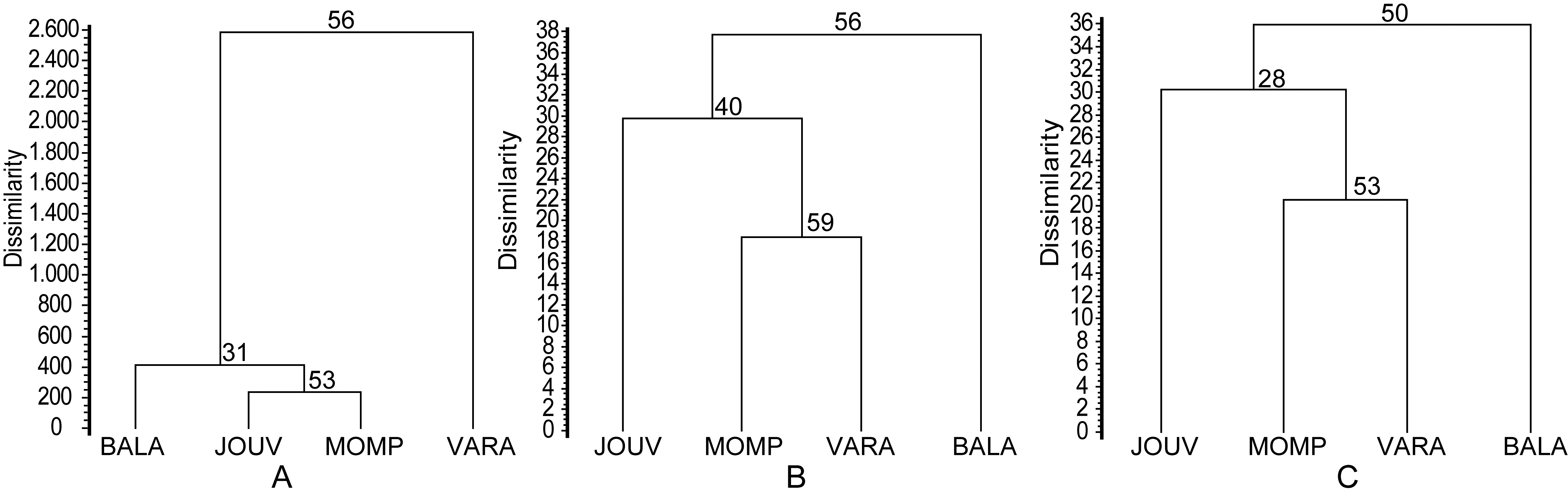

Supplement: Figure S1 — Hierarchical clustering representing the distance among the four substrates. The clustering was based on (A) the extractable fraction of cations, the distribution and representation of (B) ITS1 OTUs and (C) ITS2 OTUs within the four sites. See Materials and Methods section for the details of the analyses. (TIF) [file pone.0044233.s001.tif]

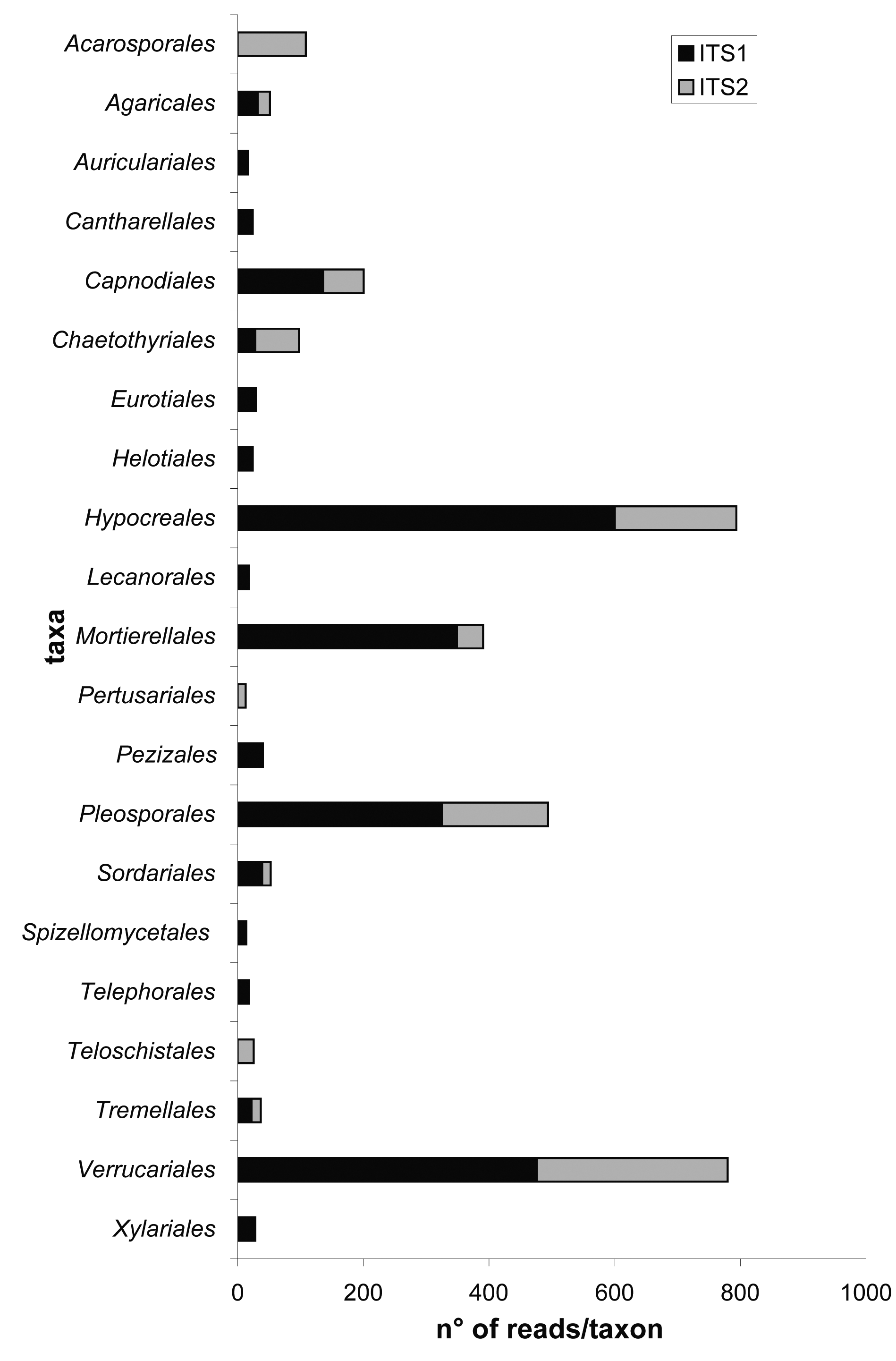

Supplement: Figure S2 — ITS1 and ITS2 blast results at genus level. The OTUs≥10 reads were grouped according to their taxon assignment at genus level and the number of ITS1 and ITS2 reads supporting each taxon is reported. (TIF) [file pone.0044233.s002.tif]
