# Supplementary material for: Fungal Diversity Is Not Determined by Mineral and Chemical Differences in Serpentine Substrates
Source: PLoS One. 2012 Sep 20;7(9):e44233. doi: 10.1371/journal.pone.0044233 (PMC3447857; doi:10.1371/journal.pone.0044233)
Supplement: Table S1 — Dominant taxa according to the ITS1 region sequencing. (DOC) [file pone.0044233.s003.doc]

**Table S1. Dominant taxa according to the ITS1 region sequencing.**

| **Kingdom** | **Phylum** | **Class** | **Order** | **Family** | **Genus** | **Specie** | **%** | **JOUV** | **MOMP** | **VARA** | **BALA** |
| --- | --- | --- | --- | --- | --- | --- | --- | --- | --- | --- | --- |
| Fungi (59) | Ascomycota (45) | Dothideomycetes (10) | Capnodiales (3) | Davidiellaceae | Cladosporium (1) |  | 1.43 | 25 | 9 | 10 | 27 |
|  |  |  |  |  | | | 1.31 | 65 | 0 | 0 | 0 |
|  |  |  | Pleosporales (7) | Pleosporaceae | Alternaria |  | 0.73 | 14 | 5 | 4 | 13 |
|  |  |  |  |  | Phoma |  | 2.04 | 25 | 4 | 72 | 0 |
|  |  |  |  |  | | | 3.80 | 0 | 55 | 94 | 39 |
|  |  |  | | | | | 0.97 | 1 | 25 | 22 | 0 |
|  |  | Eurotiomycetes (11) | Chaetothyriales | Herpotrichiellaceae | Rhinocladiella (1) |  | 0.57 | 0 | 18 | 1 | 9 |
|  |  |  | Eurotiales | Trichocomaceae | Penicillium (1) |  | 0.59 | 3 | 2 | 24 | 0 |
|  |  |  | Verrucariales | Verrucariaceae (10) | Verrucaria (2) |  | 2.14 | 17 | 4 | 85 | 0 |
|  |  |  |  |  | | | 7.47 | 2 | 0 | 0 | 368 |
|  |  | Lecanoromycetes (1) | Lecanorales | Lecanorinae |  | | 0.36 | 0 | 0 | 0 | 18 |
|  |  |  |  | | | | 0.34 | 0 | 0 | 17 | 0 |
|  |  | Leotiomycetes (2) | Helotiales (2) | Helotiaceae | Articulospora | proliferata | 0.22 | 1 | 4 | 6 | 0 |
|  |  |  |  |  | Tetracladium |  | 0.26 | 8 | 2 | 3 | 0 |
|  |  |  |  | Geomyces |  | | 1.58 | 74 | 1 | 2 | 1 |
|  |  | Pezizomycetes | Pezizales | Pyronemataceae | Geopora (1) | arenicola | 0.61 | 0 | 0 | 0 | 30 |
|  |  |  |  |  |  |  | 0.20 | 10 | 0 | 0 | 0 |
|  |  | Sordariomycetes (14) | Hypocreales (12) | Nectriaceae | Fusarium (4) |  | 4.06 | 20 | 83 | 82 | 16 |
|  |  |  |  |  | | | 7.75 | 374 | 2 | 6 | 2 |
|  |  |  |  | Cordycipitaceae | Cordyceps |  | 0.30 | 9 | 1 | 0 | 5 |
|  |  |  | Sordariales (1) | Lasiosphaeriaceae (0) |  | | 0.24 | 7 | 5 | 0 | 0 |
|  |  |  |  |  | Podospora | communis | 0.22 | 0 | 0 | 0 | 11 |
|  |  |  |  |  | | | 0.32 | 0 | 0 | 11 | 5 |
|  |  |  | Xylariales | Amphisphaeriaceae | Truncatella | angustata | 0.57 | 0 | 1 | 27 | 0 |
|  |  |  |  | Plectosphaerellaceae | Verticillium |  | 0.63 | 0 | 26 | 5 | 0 |
|  |  |  | | | | | 7 | 62 | 146 | 110 | 8 |
|  | Basidiomycota (2) | Agaricomycetes (2) | Agaricales | Agaricaceae | Coprinus |  | 0.65 | 0 | 0 | 0 | 32 |
|  |  |  | Auriculariales |  | Sebacina | vermifera | 0.34 | 0 | 0 | 17 | 0 |
|  |  |  | Cantharellales | Ceratobasidiaceae |  | | 0.48 | 12 | 0 | 12 | 0 |
|  |  |  | Telephorales | Thelephoraceae |  | | 0.36 | 0 | 0 | 0 | 18 |
|  |  | Tremellomycetes | Tremellales | Tremellaceae | Filobasidiella |  | 0.44 | 22 | 0 | 0 | 0 |
|  | Chitridiomycota | Chytridiomycetes | Spizellomycetales | Spizellomycetaceae | Spizellomyces | pseudodichotomus | 0.28 | 0 | 2 | 9 | 3 |
|  | Zigomycota (7) |  | Mortierellales | Mortierellaceae | Mortierella (7) | alpina (5) | 4.99 | 104 | 86 | 31 | 26 |
|  |  |  |  |  |  |  | 2.06 | 84 | 2 | 9 | 7 |
|  |  |  | | | | | 0.22 | 8 | 0 | 0 | 3 |
|  |  | | | | | | 3.43 | 52 | 34 | 15 | 69 |
| No hits found (11) |  | | | | | | 11.21 | 74 | 170 | 135 | 176 |
| Non fungi (0) |  | | | | | | 0.24 | 1 | 0 | 0 | 11 |
| **OTUs >10 reads** |  | | | | | | **70.01** | **1074** | **687** | **809** | **897** |
| **OTUs <10 reads** |  | | | | | | **29.99** | **283** | **394** | **518** | **290** |
| **ALL** |  | | | | | | **100** | **1357** | **1081** | **1327** | **1187** |

OTUs supported by at least 10 reads were named by BlastN matching and grouped if belonging to the same taxon.

^a^The total abundance (i.e.: number of reads supporting the taxon/total number of reads %) is reported in brackets or in the column “%”.

^b^The four columns on the right report the number of reads supporting each taxon in each site.
