# Supplementary material for: Fungal Diversity Is Not Determined by Mineral and Chemical Differences in Serpentine Substrates
Source: PLoS One. 2012 Sep 20;7(9):e44233. doi: 10.1371/journal.pone.0044233 (PMC3447857; doi:10.1371/journal.pone.0044233)
Supplement: Table S2 — Dominant taxa according to the ITS2 region sequencing. (DOC) [file pone.0044233.s004.doc]

**Table S2. Dominant taxa according to the ITS2 region sequencing.**

| **Kingdom** | **Phylum** | **Class** | **Order** | **Family** | **Genus** | **Specie** | **%** | **JOUV** | **MOMP** | **VARA** | **BALA** |
| --- | --- | --- | --- | --- | --- | --- | --- | --- | --- | --- | --- |
| Fungi (56) | Ascomycota (46) | Dothideomycetes (10) | Capnodiales |  | | | 2.40 | 30 | 16 | 6 | 13 |
|  |  |  | Pleosporales (6) | Pleosporaceae | Alternaria | alternata | 0.37 | 1 | 2 | 1 | 6 |
|  |  |  |  |  | Phoma |  | 1.77 | 0 | 22 | 26 | 0 |
|  |  |  |  |  | | | 4.10 | 7 | 38 | 52 | 14 |
|  |  |  |  | | | | 1.00 | 0 | 0 | 27 | 0 |
|  |  | Eurotiomycetes (14) | Chaetothyriales (3) |  | | | 2.14 | 22 | 35 | 0 | 1 |
|  |  |  |  | Herpotrichiellaceae | Rhinocladiella |  | 0.44 | 0 | 11 | 0 | 1 |
|  |  |  | Verrucariales | Verrucariaceae (11) | Dermatocarpon |  | 9.97 | 17 | 16 | 23 | 214 |
|  |  |  |  |  | Verrucaria |  | 0.89 | 0 | 1 | 23 | 0 |
|  |  |  |  |  |  | | 0.37 | 0 | 0 | 0 | 10 |
|  |  | Lecanoromycetes (5) | Acarosporales | Acarosporaceae (4) | Acarospora |  | 0.89 | 0 | 0 | 24 | 0 |
|  |  |  |  |  | Sarcogyne | regularis | 3.14 | 0 | 85 | 0 | 0 |
|  |  |  | Pertusariales | Megasporaceae | Aspicilia |  | 0.48 | 0 | 0 | 13 | 0 |
|  |  |  | Teloschistales | Physciaceae | Physcia |  | 0.96 | 0 | 0 | 26 | 0 |
|  |  | Leotiomycetes |  | | Geomyces |  | 0.96 | 26 | 0 | 0 | 0 |
|  |  | Sordariomycetes (11) | Hypocreales (7) | Nectriaceae | Fusarium (2) | oxysporum | 0.66 | 0 | 0 | 18 | 0 |
|  |  |  |  |  |  |  | 1.22 | 6 | 20 | 3 | 4 |
|  |  |  |  |  | Geosmithia |  | 4.88 | 129 | 0 | 2 | 1 |
|  |  |  |  |  | | | 0.41 | 0 | 0 | 11 | 0 |
|  |  |  | Sordariales |  | | | 0.52 | 0 | 0 | 3 | 11 |
|  |  |  |  | Plectosphaerellaceae | Verticillium |  | 2.99 | 5 | 64 | 12 | 0 |
|  |  |  | | | | | 5.02 | 36 | 71 | 0 | 29 |
|  | Basidiomycota (1) | Agaricomycetes | Agaricales |  | | | 0.74 | 0 | 0 | 0 | 20 |
|  |  | Tremellomycetes | Tremellales | Tremellaceae | Filobasidiella |  | 0.55 | 15 | 0 | 0 | 0 |
|  | Zigomycota |  | Mortierellales | Mortierellaceae | Mortierella (2) | alpina | 1.03 | 22 | 1 | 0 | 5 |
|  |  |  |  |  |  |  | 0.52 | 14 | 0 | 0 | 0 |
|  |  | | | | | | 7.61 | 8 | 18 | 26 | 154 |
| No hits found (3) |  | | | | | | 2.66 | 3 | 29 | 12 | 28 |
| Non fungi (1) |  | | | | | | 0.96 | 0 | 0 | 25 | 1 |
| **OTUs >10 reads** |  | | | | | | **59.66** | **341** | **429** | **333** | **512** |
| **OTUs <10 reads** |  | | | | | | **40.34** | **216** | **291** | **367** | **218** |
| **ALL** |  | | | | | | **100** | **557** | **720** | **700** | **730** |

OTUs supported by at least 10 reads were named by BlastN matching and grouped if belonging to the same taxon.

^a^The total abundance (i.e.: number of reads supporting the taxon/total number of reads %) is reported in brackets or in the column “%”.

^b^The four columns on the right report the number of reads supporting each taxon in each site.
