# Supplementary material for: Fungal Diversity Is Not Determined by Mineral and Chemical Differences in Serpentine Substrates
Source: PLoS One. 2012 Sep 20;7(9):e44233. doi: 10.1371/journal.pone.0044233 (PMC3447857; doi:10.1371/journal.pone.0044233)
Supplement: Table S3 — ITS1 dominant OTUs. OTUs supported by at least 10 reads were ranked according to their abundance. (DOC) [file pone.0044233.s005.doc]

**Table S3.** ITS1 dominant (i.e. supported by ≥10 reads) OTUs ranked according to their abundance (N° of reads supporting the OTU/total N° of reads %).

| **ITS1 OTU** | **JOUV** | **MOMP** | **VARA** | **BALA** | **tot** | **rank/abundance %** | **hit id** | **identity** | **score** | **e-value** | **hit description** | **best identification** |
| --- | --- | --- | --- | --- | --- | --- | --- | --- | --- | --- | --- | --- |
| **1** | 2 | 0 | 0 | 368 | 370 | 7.5 | gi|238058350|gb|FJ664879.1| | 97.3 | 65 | 2E-28 | Verrucaria sp. | Verrucariaceae |
| **2** | 307 | 0 | 3 | 0 | 310 | 6.3 | gi|193246449|gb|EU687056.1| | 94.3 | 68 | 3E-30 | Fungal endophyte isolate | Hypocreales |
| **3** | 14 | 109 | 20 | 0 | 143 | 2.9 | No hits found |  |  |  |  |  |
| **4** | 15 | 82 | 31 | 4 | 132 | 2.7 | gi|6706195|emb|AJ279478.1| | 98.6 | 206 | 1E-112 | Fusarium sp. | Fusarium sp. |
| **5** | 91 | 4 | 9 | 26 | 130 | 2.6 | gi|11121250|emb|AJ271630.1| | 99.4 | 164 | 1E-87 | Mortierella alpina | Mortierella alpina |
| **6** | 84 | 2 | 9 | 7 | 102 | 2.1 | gi|312434575|gb|HQ608097.1| | 100 | 145 | 3E-76 | Mortierella sp. | Mortierella sp. |
| **7** | 0 | 0 | 0 | 96 | 96 | 1.9 | No hits found |  |  |  |  |  |
| **8** | 0 | 4 | 85 | 0 | 89 | 1.8 | gi|166159481|gb|EU249486.1| | 91.8 | 114 | 1E-57 | Verrucaria muralis | Verrucaria sp. |
| **9** | 1 | 82 | 0 | 0 | 83 | 1.7 | gi|32264973|gb|AY310443.1| | 97.2 | 144 | 1E-75 | Mortierella alpina | Mortierella alpina |
| **10** | 0 | 13 | 67 | 0 | 80 | 1.6 | gi|66990820|emb|AJ972865.1| | 100 | 140 | 2E-73 | Phoma sp. | Pleosporales |
| **11** | 74 | 1 | 2 | 1 | 78 | 1.6 | gi|33622362|gb|AY345347.1| | 100 | 172 | 2E-92 | Geomyces sp. | Geomyces sp. |
| **12** | 0 | 3 | 70 | 0 | 73 | 1.5 | gi|305682535|dbj|AB488489.1| | 96.3 | 182 | 3E-98 | Phoma fimeti | Phoma sp. |
| **13** | 65 | 0 | 0 | 0 | 65 | 1.3 | gi|55783615|gb|AY805548.1| | 94.1 | 120 | 2E-61 | Capnodium sp. | Capnodiales |
| **14** | 0 | 6 | 16 | 39 | 61 | 1.2 | gi|6706174|emb|AJ279448.1| | 100 | 142 | 2E-74 | Epicoccum nigrum | Pleosporales |
| **15** | 4 | 1 | 51 | 1 | 57 | 1.2 | gi|1122874|emb|X94169.1| | 99.3 | 141 | 6E-74 | Fusarium redolens | Fusarium sp. |
| **16** | 56 | 0 | 0 | 0 | 56 | 1.1 | No hits found |  |  |  |  |  |
| **17** | 0 | 0 | 53 | 0 | 53 | 1.1 | No hits found |  |  |  |  |  |
| **18** | 0 | 51 | 0 | 0 | 51 | 1.0 | No hits found |  |  |  |  |  |
| **19** | 20 | 28 | 0 | 1 | 49 | 1.0 | gi|313799809|gb|HQ599584.1| | 94.2 | 53 | 4E-21 | Strelitziana africana | Ascomycota |
| **20** | 0 | 1 | 1 | 46 | 48 | 1.0 | gi|20531607|gb|AF502800.1| | 97.1 | 30 | 2E-07 | Leaf litter ascomycete | Fungi |
| **21** | 15 | 6 | 8 | 15 | 44 | 0.9 | gi|13539171|emb|AJ300334.1| | 98.7 | 147 | 2E-77 | Cladosporium cladosporioides | Cladosporium sp. |
| **22** | 13 | 24 | 0 | 0 | 37 | 0.7 | gi|20452220|gb|AF444494.1| | 90.9 | 31 | 3E-08 | Kurtzmanomyces nectairei | Fungi |
| **23** | 14 | 5 | 4 | 13 | 36 | 0.7 | gi|7208647|emb|AJ276055.1| | 100 | 164 | 1E-87 | Alternaria alternata | Alternaria sp. |
| **24** | 0 | 21 | 14 | 0 | 35 | 0.7 | gi|300676324|gb|GU934498.1| | 93.7 | 67 | 1E-29 | Spicellum roseum | Ascomycota |
| **25** | 12 | 0 | 22 | 0 | 34 | 0.7 | gi|32264973|gb|AY310443.1| | 97.2 | 144 | 1E-75 | Mortierella alpina | Mortierella alpina |
| **26** | 31 | 1 | 1 | 0 | 33 | 0.7 | gi|95140277|emb|AM262388.1| | 97.2 | 32 | 6E-09 | Cf. Acremonium sp. | Hypocreales |
| **27** | 0 | 0 | 0 | 32 | 32 | 0.6 | gi|19070324|gb|AF345823.1| | 94 | 138 | 5E-72 | Coprinopsis atramentaria | Coprinus |
| **28** | 0 | 0 | 31 | 0 | 31 | 0.6 | gi|66990768|emb|AJ972819.1| | 95.9 | 41 | 3E-14 | Ascomycete sp. | Ascomycota |
| **29** | 0 | 26 | 5 | 0 | 31 | 0.6 | gi|55783663|gb|AY805596.1| | 92.5 | 96 | 4E-47 | Verticillium sp. | Verticillium sp. |
| **30** | 0 | 0 | 0 | 30 | 30 | 0.6 | gi|209412931|emb|FM206433.1| | 100 | 224 | 1E-123 | Geopora arenicola | Geopora arenicola |
| **31** | 0 | 16 | 13 | 0 | 29 | 0.6 | gi|8918227|dbj|AB019364.1| | 93.8 | 56 | 4E-23 | Spicellum roseum | Ascomycota |
| **32** | 25 | 3 | 0 | 0 | 28 | 0.6 | gi|289190091|gb|GQ850385.1| | 97.3 | 33 | 3E-09 | Strelitziana africana | Fungi |
| **33** | 0 | 1 | 27 | 0 | 28 | 0.6 | gi|22773869|gb|AF405306.1| | 99.5 | 182 | 3E-98 | Truncatella angustata | Truncatella angustata |
| **34** | 25 | 1 | 2 | 0 | 28 | 0.6 | gi|237641561|gb|FJ903342.1| | 100 | 170 | 4E-91 | Ascomycota sp. | Phoma sp. |
| **35** | 10 | 3 | 2 | 12 | 27 | 0.5 | gi|15865447|emb|AJ300333.1| | 99.4 | 150 | 3E-79 | Cladosporium herbarum | Cladosporium sp. |
| **36** | 0 | 0 | 0 | 25 | 25 | 0.5 | No hits found |  |  |  |  |  |
| **37** | 25 | 0 | 0 | 0 | 25 | 0.5 | gi|193246449|gb|EU687056.1| | 94.3 | 68 | 3E-30 | Fungal endophyte isolate | Hypocreales |
| **38** | 12 | 0 | 12 | 0 | 24 | 0.5 | gi|326319947|emb|FR837929.1| | 95.7 | 148 | 5E-78 | Ceratobasidiaceae sp. | Ceratobasidiaceae |
| **39** | 0 | 24 | 0 | 0 | 24 | 0.5 | gi|56181649|gb|AY781244.1| | 93.3 | 88 | 3E-42 | Ascomycete sp. | Ascomycota |
| **40** | 0 | 23 | 0 | 0 | 23 | 0.5 | gi|46488885|gb|AY531687.1| | 95.7 | 113 | 5E-57 | Phoma fimeti | Phoma sp. |
| **41** | 0 | 1 | 21 | 0 | 22 | 0.4 | gi|27528330|emb|AJ496631.1| | 94 | 64 | 7E-28 | Phaeosphaeria phragmitis | Dothideomycetes |
| **42** | 22 | 0 | 0 | 0 | 22 | 0.4 | gi|32187794|emb|AJ568015.1| | 99.2 | 117 | 1E-59 | Filobasidiella depauperata | Filobasidiella sp. |
| **43** | 0 | 2 | 0 | 19 | 21 | 0.4 | gi|231275068|emb|FN392306.1| | 98 | 46 | 5E-17 | Fungal endophyte | Fungi |
| **44** | 20 | 0 | 0 | 0 | 20 | 0.4 | gi|194270634|gb|EU835936.1| | 99 | 187 | 1E-101 | Endophytic ascomycete sp. | Ascomycota |
| **45** | 0 | 0 | 0 | 19 | 19 | 0.4 | No hits found |  |  |  |  |  |
| **46** | 3 | 3 | 12 | 0 | 18 | 0.4 | gi|20531607|gb|AF502800.1| | 98 | 45 | 2E-16 | Leaf litter ascomycete | Ascomycota |
| **47** | 0 | 0 | 0 | 18 | 18 | 0.4 | gi|62906739|gb|DQ001303.1| | 97 | 59 | 7E-25 | Sulcaria sp. | Lecanorineae |
| **48** | 0 | 0 | 0 | 18 | 18 | 0.4 | gi|6273857|gb|AF184745.1| | 98 | 93 | 4E-45 | Thelephoraceae sp. | Thelephoraceae |
| **49** | 0 | 17 | 0 | 0 | 17 | 0.3 | gi|66796403|gb|DQ008125.1| | 93.6 | 31 | 4E-08 | Cladosporium adianticola | Ascomycota |
| **50** | 0 | 17 | 0 | 0 | 17 | 0.3 | gi|227184141|gb|FJ265763.1| | 94.7 | 59 | 7E-25 | Rhinocladiella sp. | Rhinocladiella sp. |
| **51** | 0 | 0 | 17 | 0 | 17 | 0.3 | gi|156052284|gb|EF535192.1| | 98 | 46 | 4E-17 | Candelariella rosulans | Lecanoromycetes |
| **52** | 17 | 0 | 0 | 0 | 17 | 0.3 | gi|238058347|gb|FJ664876.1| | 96.5 | 49 | 6E-19 | Verrucaria muralis | Verrucaria sp. |
| **53** | 0 | 0 | 17 | 0 | 17 | 0.3 | gi|193885585|gb|EU625995.1| | 93.2 | 60 | 2E-25 | Sebacina vermifera | Sebacina vermifera |
| **54** | 0 | 1 | 12 | 3 | 16 | 0.3 | No hits found |  |  |  |  |  |
| **55** | 2 | 1 | 12 | 1 | 16 | 0.3 | No hits found |  |  |  |  |  |
| **56** | 11 | 1 | 2 | 2 | 16 | 0.3 | gi|95140277|emb|AM262388.1| | 94.7 | 30 | 9E-08 | Cf. Acremonium sp. | Hypocreales |
| **57** | 0 | 0 | 11 | 5 | 16 | 0.3 | gi|56411543|gb|AY681199.1| | 100 | 44 | 6E-16 | Apodus deciduus | Sordariales |
| **58** | 3 | 0 | 13 | 0 | 16 | 0.3 | gi|95140230|emb|AM262346.1| | 100 | 58 | 3E-24 | Penicillium sp. | Penicillium sp. |
| **59** | 0 | 14 | 1 | 0 | 15 | 0.3 | gi|45181467|gb|AY398700.1| | 95 | 29 | 5E-07 | Lecanora straminea | Dothideomycetes |
| **60** | 9 | 1 | 0 | 5 | 15 | 0.3 | gi|15722483|emb|AJ345086.1| | 100 | 161 | 8E-86 | Beauveria tenella | Cordyceps |
| **61** | 0 | 14 | 0 | 0 | 14 | 0.3 | gi|45558599|gb|AY559355.1| | 96.1 | 39 | 8E-13 | Melanized limestone ascomycete | Ascomycota |
| **62** | 0 | 2 | 9 | 3 | 14 | 0.3 | gi|290874459|gb|GQ160458.1| | 100 | 35 | 2E-10 | Spizellomyces pseudodichotomus | Spizellomyces pseudodichotomus |
| **63** | 2 | 0 | 5 | 7 | 14 | 0.3 | No hits found |  |  |  |  |  |
| **64** | 0 | 11 | 2 | 0 | 13 | 0.3 | gi|45558599|gb|AY559355.1| | 94.1 | 147 | 2E-77 | Melanized limestone ascomycete | Ascomycota |
| **65** | 0 | 13 | 0 | 0 | 13 | 0.3 | gi|261497785|gb|GQ922919.1| | 100 | 155 | 3E-82 | Leptosphaeria sp. | Pleosporales |
| **66** | 0 | 2 | 11 | 0 | 13 | 0.3 | gi|3925742|emb|AJ010484.1| | 100 | 58 | 3E-24 | Penicillium coralligerum | Penicillium sp. |
| **67** | 8 | 2 | 3 | 0 | 13 | 0.3 | gi|85679810|gb|DQ350129.1| | 100 | 160 | 3E-85 | Tetracladium sp. | Tetracladium sp. |
| **68** | 13 | 0 | 0 | 0 | 13 | 0.3 | gi|5817610|gb|AF138814.1| | 90.5 | 59 | 5E-25 | Arthothelium spectabile | Fungi |
| **69** | 1 | 4 | 3 | 4 | 12 | 0.2 | gi|312434623|gb|HQ608145.1| | 98.2 | 151 | 8E-80 | Humicola fuscoatra | Fungi |
| **70** | 0 | 0 | 12 | 0 | 12 | 0.2 | No hits found |  |  |  |  |  |
| **71** | 0 | 1 | 0 | 11 | 12 | 0.2 | No hits found |  |  |  |  |  |
| **72** | 0 | 0 | 6 | 6 | 12 | 0.2 | gi|231275068|emb|FN392306.1| | 100 | 44 | 7E-16 | Fungal endophyte | Ascomycota |
| **73** | 7 | 5 | 0 | 0 | 12 | 0.2 | gi|261599383|gb|GQ922573.1| | 98.1 | 150 | 3E-79 | Podospora tetraspora | Lasiosphaeriaceae |
| **74** | 1 | 0 | 0 | 11 | 12 | 0.2 | gi|77456205|gb|DQ219433.1| | 100 | 153 | 5E-81 | Nigrospora oryzae | Eukaryota |
| **75** | 1 | 0 | 0 | 11 | 12 | 0.2 | gi|95136108|emb|AM262427.1| | 100 | 148 | 4E-78 | Fusarium culmorum | Fusarium sp. |
| **76** | 10 | 0 | 1 | 0 | 11 | 0.2 | gi|291498441|gb|GU566253.1| | 98.4 | 110 | 4E-55 | Bionectria ochroleuca | Ascomycota |
| **77** | 4 | 1 | 5 | 1 | 11 | 0.2 | gi|66471703|emb|AJ971454.1| | 95.1 | 29 | 7E-07 | Coniosporium sp. | Ascomycota |
| **78** | 0 | 0 | 11 | 0 | 11 | 0.2 | gi|291010787|gb|GQ499377.1| | 100 | 192 | 1E-104 | Kochiomyces sp. | Fungi |
| **79** | 0 | 0 | 0 | 11 | 11 | 0.2 | No hits found |  |  |  |  |  |
| **80** | 0 | 11 | 0 | 0 | 11 | 0.2 | gi|327391620|gb|HQ709322.1| | 97.5 | 173 | 7E-93 | Capronia peltigerae | Ascomycota |
| **81** | 0 | 7 | 1 | 3 | 11 | 0.2 | No hits found |  |  |  |  |  |
| **82** | 0 | 1 | 1 | 9 | 11 | 0.2 | gi|227184141|gb|FJ265763.1| | 96 | 63 | 3E-27 | Rhinocladiella sp. | Rhinocladiella sp. |
| **83** | 8 | 0 | 0 | 3 | 11 | 0.2 | gi|251752836|dbj|AB476411.1| | 100 | 144 | 1E-75 | Mortierella alpina | Zygomycota |
| **84** | 0 | 0 | 0 | 11 | 11 | 0.2 | gi|185869734|gb|EU621831.1| | 97.8 | 123 | 3E-63 | Podospora communis | Podospora communis |
| **85** | 1 | 4 | 6 | 0 | 11 | 0.2 | gi|198241584|gb|FJ000395.1| | 95.3 | 136 | 7E-71 | Articulospora proliferata | Articulospora proliferata |
| **86** | 1 | 10 | 0 | 0 | 11 | 0.2 | gi|95140239|emb|AM262355.1| | 100 | 139 | 9E-73 | Phoma exigua | Dothideomycetes |
| **87** | 5 | 0 | 6 | 0 | 11 | 0.2 | gi|62718901|emb|AJ890436.1| | 99.2 | 118 | 3E-60 | Phoma eupyrena | Ascomycota |
| **88** | 0 | 0 | 11 | 0 | 11 | 0.2 | gi|95140238|emb|AM262354.1| | 100 | 139 | 9E-73 | Phoma sp. | Pleosporales |
| **89** | 10 | 0 | 0 | 0 | 10 | 0.2 | gi|209412909|emb|FM206411.1| | 95.5 | 68 | 3E-30 | Geopora cervina | Geopora sp. |
| **90** | 0 | 0 | 10 | 0 | 10 | 0.2 | No hits found |  |  |  |  |  |
| **91** | 0 | 0 | 10 | 0 | 10 | 0.2 | No hits found |  |  |  |  |  |
| **92** | 0 | 0 | 10 | 0 | 10 | 0.2 | gi|27528330|emb|AJ496631.1| | 94.1 | 61 | 4E-26 | Phaeosphaeria phragmitis | Ascomycota |
| **93** | 0 | 0 | 10 | 0 | 10 | 0.2 | gi|294346397|gb|GU237746.1| | 97.9 | 128 | 3E-66 | Phoma polemonii | Ascomycota |
