# Supplementary material for: Fungal Diversity Is Not Determined by Mineral and Chemical Differences in Serpentine Substrates
Source: PLoS One. 2012 Sep 20;7(9):e44233. doi: 10.1371/journal.pone.0044233 (PMC3447857; doi:10.1371/journal.pone.0044233)
Supplement: Table S4 — ITS2 dominant OTUs. OTUs supported by at least 10 reads were ranked according to their abundance. (DOC) [file pone.0044233.s006.doc]

**Table S4.** ITS2 dominant (i.e. supported by ≥10 reads) OTUs ranked according to their abundance (N° of reads supporting the OTU/total N° of reads %).

| **ITS2 OTU** | **JOUV** | **MOMP** | **VARA** | **BALA** | **tot** | **rank/abundance %** | **hit id** | **identity** | **score** | **e-value** | **hit description** | **best identification** |
| --- | --- | --- | --- | --- | --- | --- | --- | --- | --- | --- | --- | --- |
| **1** | 17 | 16 | 23 | 214 | 270 | 9.97 | gi|18461393|gb|AF333131.1| | 91.4 | 46 | 3E-17 | Dermatocarpon americanum | Dermatocarpon sp. |
| **2** | 129 | 0 | 2 | 1 | 132 | 4.88 | gi|117164168|emb|AM181472.1| | 93.3 | 66 | 3E-29 | Geosmithia sp. | Geosmithia sp. |
| **3** | 7 | 27 | 52 | 11 | 97 | 3.58 | gi|6706174|emb|AJ279448.1| | 100 | 110 | 1E-55 | Epicoccum nigrum | Pleosporales |
| **4** | 0 | 0 | 0 | 85 | 85 | 3.14 | gi|231275068|emb|FN392306.1| | 98.2 | 103 | 2E-51 | Fungal endophyte | Fungi |
| **5** | 0 | 85 | 0 | 0 | 85 | 3.14 | gi|85819899|gb|AY853393.2| | 97.5 | 106 | 4E-53 | Sarcogyne regularis | Sarcogyne regularis |
| **6** | 5 | 64 | 12 | 0 | 81 | 2.99 | gi|55783663|gb|AY805596.1| | 99.1 | 103 | 2E-51 | Verticillium sp. | Verticillium sp. |
| **7** | 3 | 3 | 19 | 36 | 61 | 2.25 | gi|231275068|emb|FN392306.1| | 97.3 | 99 | 5E-49 | Fungal endophyte | Fungi |
| **8** | 0 | 22 | 26 | 0 | 48 | 1.77 | gi|118918356|emb|AM236587.1| | 98.3 | 109 | 6E-55 | Phoma sp. | Phoma sp. |
| **9** | 0 | 43 | 0 | 0 | 43 | 1.59 | gi|241914484|gb|FJ911882.1| | 96.2 | 85 | 1E-40 | Mycocentrospora sp. | Ascomycota |
| **10** | 4 | 16 | 6 | 13 | 39 | 1.44 | gi|13539172|emb|AJ300335.1| | 100 | 131 | 5E-68 | Cladosporium cladosporioides | Capnodiales |
| **11** | 6 | 20 | 3 | 4 | 33 | 1.22 | gi|189047008|dbj|AB369460.1| | 99.2 | 110 | 2E-55 | Fusarium tricinctum | Fusarium sp. |
| **12** | 1 | 29 | 1 | 0 | 31 | 1.15 | No hits found |  |  |  |  |  |
| **13** | 0 | 0 | 0 | 29 | 29 | 1.07 | gi|304651454|gb|HQ115712.1| | 97.3 | 100 | 1E-49 | Lecythophora sp. | Ascomycota |
| **14** | 22 | 1 | 0 | 5 | 28 | 1.03 | gi|145977444|gb|EF202182.1| | 99.1 | 106 | 3E-53 | Mortierella alpina | Mortierella alpina |
| **15** | 0 | 0 | 27 | 0 | 27 | 1.00 | gi|83627184|emb|AM176737.1| | 92.2 | 67 | 6E-30 | Phoma sp. | Dothideomycetes |
| **16** | 26 | 0 | 0 | 0 | 26 | 0.96 | gi|75265669|gb|DQ117442.1| | 100 | 119 | 7E-61 | Geomyces pannorum | Geomyces sp. |
| **17** | 0 | 0 | 26 | 0 | 26 | 0.96 | gi|13272257|gb|AF224418.1| | 99.1 | 109 | 6E-55 | Physcia dubia | Physcia sp. |
| **18** | 26 | 0 | 0 | 0 | 26 | 0.96 | gi|145453074|gb|EF191425.1| | 100 | 111 | 4E-56 | Ascomycete sp. | Capnodiales |
| **19** | 15 | 9 | 0 | 0 | 24 | 0.89 | gi|283857942|gb|FJ917556.1| | 100 | 39 | 4E-13 | Strelitziana mali | Chaetothyriales |
| **20** | 0 | 0 | 24 | 0 | 24 | 0.89 | gi|103000877|gb|DQ525519.1| | 92.9 | 81 | 3E-38 | Acarospora sp. | Acarospora sp. |
| **21** | 0 | 1 | 23 | 0 | 24 | 0.89 | gi|238058294|gb|FJ664823.1| | 90 | 60 | 1E-25 | Verrucaria csernaensis | Verrucaria sp. |
| **22** | 0 | 3 | 2 | 18 | 23 | 0.85 | gi|4206353|gb|AF050277.1| | 100 | 33 | 0.000000002 | Phaeococcomyces catenatus | Fungi |
| **23** | 21 | 0 | 0 | 0 | 21 | 0.78 | gi|6706022|emb|AJ279457.1| | 100 | 118 | 3E-60 | Ascomycete sp. | Ascomycota |
| **24** | 7 | 12 | 0 | 1 | 20 | 0.74 | gi|283857942|gb|FJ917556.1| | 100 | 39 | 4E-13 | Strelitziana mali | Chaetothyriales |
| **25** | 0 | 0 | 0 | 20 | 20 | 0.74 | gi|94556740|gb|DQ486694.1| | 93.9 | 33 | 0.000000001 | Coprinopsis atramentaria | Agaricales |
| **26** | 0 | 0 | 18 | 0 | 18 | 0.66 | gi|1805769|emb|Y07991.1| | 100 | 116 | 4E-59 | F.oxysporum | Fusarium oxysporum |
| **27** | 2 | 0 | 0 | 15 | 17 | 0.63 | gi|238058294|gb|FJ664823.1| | 89.4 | 61 | 3E-26 | Verrucaria csernaensis | No hits found |
| **28** | 1 | 15 | 0 | 0 | 16 | 0.59 | gi|327391620|gb|HQ709322.1| | 98.4 | 118 | 3E-60 | Capronia peltigerae | Ascomycota |
| **29** | 0 | 0 | 15 | 0 | 15 | 0.55 | gi|27528710|emb|AJ431679.1| | 91.6 | 75 | 1E-34 | Stichococcus mirabilis | Stichococcus mirabilis |
| **30** | 15 | 0 | 0 | 0 | 15 | 0.55 | gi|32187794|emb|AJ568015.1| | 99.2 | 119 | 7E-61 | Filobasidiella depauperata | Filobasidiella sp. |
| **31** | 5 | 4 | 5 | 1 | 15 | 0.55 | gi|231275068|emb|FN392306.1| | 95.1 | 79 | 4E-37 | Fungal endophyte | Fungi |
| **32** | 0 | 14 | 0 | 0 | 14 | 0.52 | gi|323404653|gb|HQ634649.1| | 100 | 47 | 7E-18 | Chaetothyriales sp. | Chaetothyriales |
| **33** | 0 | 11 | 0 | 3 | 14 | 0.52 | gi|238557823|gb|FJ427058.1| | 100 | 111 | 4E-56 | Phoma radicina | Pleosporales |
| **34** | 14 | 0 | 0 | 0 | 14 | 0.52 | gi|109390545|emb|AJ878504.1| | 99.1 | 104 | 5E-52 | Mortierella elongata | Mortierella sp. |
| **35** | 0 | 0 | 3 | 11 | 14 | 0.52 | gi|12583572|emb|AJ271575.1| | 95.6 | 56 | 2E-23 | Thielavia subthermophila | Sordariales |
| **36** | 14 | 0 | 0 | 0 | 14 | 0.52 | gi|194270634|gb|EU835936.1| | 100 | 102 | 8E-51 | Endophytic ascomycete sp. | Ascomycota |
| **37** | 0 | 0 | 13 | 0 | 13 | 0.48 | gi|220683818|gb|FJ532370.1| | 95.6 | 75 | 1E-34 | Aspicilia aquatica | Aspicilia sp. |
| **38** | 0 | 0 | 0 | 13 | 13 | 0.48 | No hits found |  |  |  |  |  |
| **39** | 0 | 13 | 0 | 0 | 13 | 0.48 | gi|28974812|gb|AY204588.1| | 93.1 | 42 | 5E-15 | Alatospora acuminata | Ascomycota |
| **40** | 0 | 11 | 0 | 1 | 12 | 0.44 | gi|66990748|emb|AJ972799.1| | 97.8 | 42 | 6E-15 | Rhinocladiella sp. | Rhinocladiella sp. |
| **41** | 0 | 3 | 0 | 9 | 12 | 0.44 | gi|231275068|emb|FN392306.1| | 95.5 | 86 | 3E-41 | Fungal endophyte | Fungi |
| **42** | 0 | 0 | 10 | 1 | 11 | 0.41 | gi|257792857|gb|FJ792801.1| | 99.3 | 133 | 4E-69 | Trebouxia incrustata | Trebouxia sp. |
| **43** | 0 | 0 | 11 | 0 | 11 | 0.41 | gi|134303150|gb|EF469156.1| | 97.1 | 31 | 0.00000003 | Clavascidium sp. | No hits found |
| **44** | 0 | 0 | 11 | 0 | 11 | 0.41 | gi|39753147|gb|AY378154.1| | 100 | 120 | 2E-61 | Acremonium furcatum | Hypocreales |
| **45** | 0 | 0 | 0 | 10 | 10 | 0.37 | gi|189182739|gb|EU553497.1| | 100 | 115 | 2E-58 | Polyblastia wheldonii | Verrucariaceae |
| **46** | 1 | 2 | 1 | 6 | 10 | 0.37 | gi|7208647|emb|AJ276055.1| | 100 | 114 | 6E-58 | Alternaria alternata | Alternaria alternata |
| **47** | 0 | 5 | 0 | 5 | 10 | 0.37 | gi|231275068|emb|FN392306.1| | 92.8 | 51 | 2E-20 | Fungal endophyte | Fungi |
