# Supplementary material for: Fungal Diversity Is Not Determined by Mineral and Chemical Differences in Serpentine Substrates
Source: PLoS One. 2012 Sep 20;7(9):e44233. doi: 10.1371/journal.pone.0044233 (PMC3447857; doi:10.1371/journal.pone.0044233)
Supplement: Table S5 — Primers used in the described experiments. (DOC) [file pone.0044233.s007.doc]

**Table S5**. Primers used in the described experiments.

| ITS1F | 5’- CTTGGTCATTTAGAGGAAGTAA-3’ |
| --- | --- |
| ITS1F-GC | 5’- CTTGGTCATTTAGAGGAAGTAA-[GC]20 -3’ |
| ITS1F-adaptA | 5’- GCCTCCCTCGCGCCATCAGCTTGGTCATTTAGAGGAAGTAA-3’ |
| ITS1FAA-A | 5’- GCCTCCCTCGCGCCATCAG**AA**CTTGGTCATTTAGAGGAAGTAA-3’ |
| ITS2 | 5’- GCTGCGTTCTTCATCGATGC-3’ |
| ITS2-adaptB | 5’- GCCTTGCCAGCCCGCTCAGGCTGCGTTCTTCATCGATGC-3’ |
| ITS3-adaptA | 5’- GCCTCCCTCGCGCCATCAGGCATCGATGAAGAACGCAGC-3’ |
| ITS3TC-adaptA | 5’- GCCTCCCTCGCGCCATCAG**TC**GCATCGATGAAGAACGCAGC-3’ |
| ITS4 | 5’- TCCTCCGCTTATTGATATGC-3’ |
| ITS4-adaptB | 5’ - GCCTTGCCAGCCCGCTCAGTCCTCCGCTTATTGATATGC-3’ |
